# Endothelial cell‐derived angiopoietin‐2 is a therapeutic target in treatment‐naive and bevacizumab‐resistant glioblastoma

**DOI:** 10.15252/emmm.201505505

**Published:** 2015-12-14

**Authors:** Alexander Scholz, Patrick N Harter, Sebastian Cremer, Burak H Yalcin, Stefanie Gurnik, Maiko Yamaji, Mariangela Di Tacchio, Kathleen Sommer, Peter Baumgarten, Oliver Bähr, Joachim P Steinbach, Jörg Trojan, Martin Glas, Ulrich Herrlinger, Dietmar Krex, Matthias Meinhardt, Astrid Weyerbrock, Marco Timmer, Roland Goldbrunner, Martina Deckert, Christian Braun, Jens Schittenhelm, Jochen T Frueh, Evelyn Ullrich, Michel Mittelbronn, Karl H Plate, Yvonne Reiss

**Affiliations:** ^1^ Institute of Neurology (Edinger Institute)Goethe University Medical SchoolFrankfurtGermany; ^2^ German Cancer Consortium (DKTK)Partner Site Frankfurt/MainzFrankfurtGermany; ^3^ Department of NeurosurgeryGoethe University Medical SchoolFrankfurtGermany; ^4^ Senckenberg Institute of NeurooncologyGoethe University Medical SchoolFrankfurtGermany; ^5^ Medical Clinic IGoethe University Medical SchoolFrankfurtGermany; ^6^ Klinische Kooperationseinheit NeuroonkologieRobert Janker KlinikBonnGermany; ^7^ Neurologische Universitätsklinik BonnBonnGermany; ^8^ Klinik und Poliklinik für NeurochirurgieUniversitätsklinikum Carl Gustav CarusDresdenGermany; ^9^ Institut für PathologieUniversitätsklinikum Carl Gustav CarusDresdenGermany; ^10^ Klinik für NeurochirurgieUniversitätsklinikum FreiburgFreiburgGermany; ^11^ Zentrum für NeurochirurgieUniklinik KölnKölnGermany; ^12^ Institut für NeuropathologieUniklinik KölnKölnGermany; ^13^ Zentrum für NeuroonkologieUniversitätsklinik TübingenTübingenGermany; ^14^ Abteilung NeuropathologieUniversitätsklinik TübingenTübingenGermany; ^15^ LOEWE Center for Cell and Gene TherapyGoethe University Medical SchoolFrankfurtGermany; ^16^ Pediatric Hematology & OncologyChildren's HospitalGoethe University Medical SchoolFrankfurtGermany; ^17^ Laboratory of Immunology and Vascular BiologyDepartment of PathologyStanford University School of MedicineStanfordCAUSA

**Keywords:** anti‐angiogenic therapy, glioblastoma, macrophage polarization, therapy resistance, tumor angiogenesis, Cancer, Neuroscience, Pharmacology & Drug Discovery

## Abstract

Glioblastoma multiforme (GBM) is treated by surgical resection followed by radiochemotherapy. Bevacizumab is commonly deployed for anti‐angiogenic therapy of recurrent GBM; however, innate immune cells have been identified as instigators of resistance to bevacizumab treatment. We identified angiopoietin‐2 (Ang‐2) as a potential target in both naive and bevacizumab‐treated glioblastoma. Ang‐2 expression was absent in normal human brain endothelium, while the highest Ang‐2 levels were observed in bevacizumab‐treated GBM. In a murine GBM model, VEGF blockade resulted in endothelial upregulation of Ang‐2, whereas the combined inhibition of VEGF and Ang‐2 leads to extended survival, decreased vascular permeability, depletion of tumor‐associated macrophages, improved pericyte coverage, and increased numbers of intratumoral T lymphocytes. CD206^+^ (M2‐like) macrophages were identified as potential novel targets following anti‐angiogenic therapy. Our findings imply a novel role for endothelial cells in therapy resistance and identify endothelial cell/myeloid cell crosstalk mediated by Ang‐2 as a potential resistance mechanism. Therefore, combining VEGF blockade with inhibition of Ang‐2 may potentially overcome resistance to bevacizumab therapy.

## Introduction

Glioblastoma multiforme (GBM) is the most frequent primary malignant brain tumor in adults (Ohgaki & Kleihues, 2005). Standard therapy includes surgical resection, followed by treatment with temozolomide radiochemotherapy (Stupp *et al*, 2005). Since the first approval of bevacizumab for treatment of metastatic colorectal cancer (Hurwitz *et al*, 2004), anti‐angiogenic therapy targeting the VEGF signaling pathway has been approved for a number of cancer entities (Carmeliet & Jain, 2011; Welti *et al*, 2013). Currently, bevacizumab is widely used for the treatment of recurrent GBM (Cohen *et al*, 2009). Although two phase III clinical trials reported prolonged progression‐free survival in primary GBM, beneficial results appear to be transient as tumors eventually progress and the majority of patients does not benefit from an increased overall survival (Chinot *et al*, 2014; Gilbert *et al*, 2014). Of note, based on expression profiling, GBMs have been subdivided in different genetic subtypes (Phillips *et al*, 2006; Verhaak *et al*, 2010), and a recent study suggests that only glioblastomas that display the proneural subtype are susceptible to bevacizumab therapy (Sandmann *et al*, 2015). Additional radiological, tissue‐ or blood‐based biomarkers that predict responses to bevacizumab therapy are largely missing (Brauer *et al*, 2013).

VEGF‐induced “accessory” cells have been recognized as major contributors to angiogenesis through the secretion of numerous cytokines and growth factors (Grunewald *et al*, 2006; Avraham‐Davidi *et al*, 2013). At present, several studies attributed the resistance to bevacizumab therapy to the rebound of innate immune cells that may foster tumor growth (Shojaei *et al*, 2007; Chung *et al*, 2013). Therefore, novel drug regimens that combine VEGF blockade with other types of therapy are currently under intense investigation. Notably, a recent randomized phase II trial in patients with recurrent GBM pre‐treated with temozolomide radiochemotherapy reported a significant advantage in 9‐month overall survival in patients that received lomustine plus bevacizumab, compared to patients that received either drug alone (Taal *et al*, 2014). These findings suggest that the combination of VEGF blockade with chemotherapy or targeted therapy may be superior to anti‐angiogenic monotherapy.

In line with these observations, drugs targeting angiopoietin signaling have been tested in preclinical models, either as monotherapy (Oliner *et al*, 2004; Falcón *et al*, 2009; Coxon *et al*, 2010; Huang *et al*, 2011; Mazzieri *et al*, 2011; Holopainen *et al*, 2012; Leow *et al*, 2012; Thomas *et al*, 2013) or in combination with VEGF therapy (Brown *et al*, 2010; Hashizume *et al*, 2010; Koh *et al*, 2010; Daly *et al*, 2013; Kienast *et al*, 2013; Rigamonti *et al*, 2014). Among the drugs targeting angiopoietin signaling, a peptibody that blocks Ang‐2 (and to a lesser extent Ang‐1) is currently being evaluated in clinical phase III for ovarian cancer (Liontos *et al*, 2014; Monk *et al*, 2014). In conjunction with VEGF and its receptors, the angiopoietin/Tie system is fundamental for blood vessel growth (Augustin *et al*, 2009; Eklund & Saharinen, 2013; Reiss *et al*, 2015; Scholz *et al*, 2015). Angiopoietin signaling critically drives angiogenesis and remodeling during cancer progression. Ang‐2 is specifically upregulated under angiogenic conditions and thus hardly detectable in the healthy vasculature (Stratmann *et al*, 1998; Holash *et al*, 1999). As such, it provides an ideal target for tumor therapy.

A number of years ago, we described the specific upregulation of Ang‐2 in newly formed GBM vessels (Stratmann *et al*, 1998). More recently, we provided evidence that in addition to its function in the vascular compartment, Ang‐2 also affects the recruitment of innate immune cells (Scholz *et al*, 2011). By applying a transgenic mouse model with targeted expression of Ang‐2 in the vasculature (Reiss *et al*, 2007), we observed infiltration of myeloid cells in multiple organs in a β2‐integrin‐dependent manner (Scholz *et al*, 2011). This effect was observed upon long‐term Ang‐2 expression in transgenic mice but also in pathological settings such as inflammation or cancer (Coffelt *et al*, 2010; Scholz *et al*, 2011). Moreover, tumor‐infiltrating myeloid cells were identified to be polarized toward the pro‐angiogenic M2 phenotype in the presence of Ang‐2 (Coffelt *et al*, 2010). Those cells had the capacity to prevent immune cell activation and to promote the expansion of T regulatory cells in mouse tumors (Coffelt *et al*, 2011). We thus reasoned that specific elimination of myeloid cells by inhibition of Ang‐2 would be detrimental for tumor progression in preclinical cancer models. We hypothesized that combined anti‐VEGF and anti‐Ang‐2 therapy would affect the vasculature and stimulate the host immune system as well.

In this study, we aimed to investigate whether combined anti‐VEGF/anti‐Ang‐2 therapy is superior for the treatment of brain cancer when compared to the inhibition of either signaling pathway alone. To test such hypothesis, we applied immunocompetent preclinical models of glioblastoma (using GL261 and GL261‐luc cells) and designed single and combination therapies with AMG386 (Trebananib, a first‐in‐class Ang‐1/2 neutralizing peptibody) and aflibercept (Zaltrap^®^, also known as VEGF‐trap that blocks VEGF‐A, VEGF‐B, and placenta growth factor (PlGF; see Materials and Methods) (Holash *et al*, 2002; Coxon *et al*, 2010). Although the combined targeting of VEGF and Ang‐2 has been described previously (Brown *et al*, 2010; Hashizume *et al*, 2010; Koh *et al*, 2010b; Daly *et al*, 2013; Gerald *et al*, 2013; Kienast *et al*, 2013; Rigamonti *et al*, 2014), this is the first study that explores the potential of dual anti‐angiogenic therapy in orthotopic brain tumors. Moreover, we provide evidence that the combined inhibition of angiopoietin and VEGF signaling may obliterate resistance to VEGF monotherapy caused by upregulation of Ang‐2 in endothelial cells, accompanied by the presence of alternatively polarized perivascular macrophages.

## Results

### Ang‐2 expression correlates with WHO grading and represents a therapeutic target in human gliomas

Ang‐2 is an angiogenic growth factor that plays a key role at early stages of tumor progression where it drives vessel remodeling, cooption, and angiogenic sprouting (Eklund & Saharinen, 2013; Scholz *et al*, 2015). Ang‐2 is rapidly released and is easily detected in the serum of patients with neoplastic and inflammatory diseases (Park *et al*, 2007; Helfrich *et al*, 2009; Sallinen *et al*, 2014).

**Figure 1:**
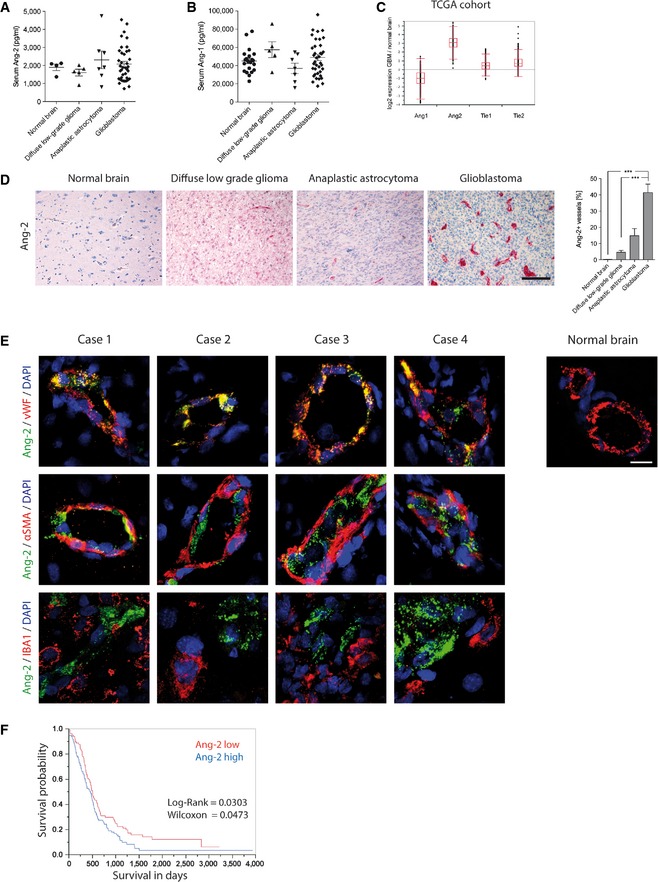
Endothelial Ang‐2 upregulation correlates with WHO grading in human gliomas A, B
ELISA displaying human Ang‐2 (A) and Ang‐1 (B) level in serum of healthy volunteers (Ang‐2 *n* =* *4; Ang‐1 *n* =* *21) or patients with low‐grade diffuse glioma (WHO II) (*n *=* *5), anaplastic astrocytoma (WHO III) (*n *=* *7), or glioblastoma (WHO IV) (*n *=* *39) are shown.CThe TCGA database was accessed to obtain gene expression level for Ang‐1, Ang‐2, Tie2, and Tie1 in GBM in comparison with normal brain (box‐and whisker plot showing median, 25–75^th^ percentile, upper and lower quartile including outliers).DAng‐2 expression and quantitative analysis of Ang‐2 in healthy human brain (*n *=* *3), low‐grade diffuse glioma (*n *=* *14), anaplastic astrocytoma (*n *=* *12), or glioblastoma (*n *=* *11) are shown. Scale bar: 100 μm.ECo‐staining of Ang‐2 and vWF (endothelial cells), αSMA (mural cells), and IBA1 (microglia) in different glioblastoma specimen. Normal brain tissue was used to assess Ang‐2 staining specificity. Scale bar: 20 μm.FAng‐2 predicts survival of glioblastoma patients.Data information: If not indicated differently, Kruskal–Wallis test (Dunn's post‐test) was applied, ***P < 0.005*;* data are mean ± SEM.Source data are available online for this figure.

To date, the relevance of Ang‐2 as a potential prognostic marker in brain malignancies has not been evaluated. Therefore, we screened the serum of glioma patients (low‐grade diffuse glioma/WHO grade II, anaplastic astrocytoma/WHO grade III, and glioblastoma/WHO grade IV) for Ang‐2 expression. Compared to healthy controls, Ang‐2 serum levels increased slightly across WHO grades (Fig 1A) where as expression levels of Ang‐1, which typically are not upregulated in cancer patients, remained unchanged (Fig 1B). In a separate cohort of GBM patients (*n* = 19), we examined serum Ang‐2 levels at three different time points, namely prior to bevacizumab therapy, during bevacizumab therapy (best response) and at tumor progression. Serum levels of Ang‐2 did not change during therapy (median levels were 2,006 ng/ml prior to bevacizumab therapy versus 1,937 ng/ml at best response, versus 1,761 ng/ml at progression, n.s.).

Expression data obtained from The Cancer Genome Atlas (TCGA) confirmed the upregulation of Ang‐2 in human glioblastoma compared to normal brain (Fig 1C; *N* = 553; https://tcga-data.nci.nih.gov/tcga/). In contrast, Ang‐1, Tie1, and Tie2 levels were much closer to normal human brain. These findings support the notion that tuning of Tie signaling in glioblastoma mainly occurs via upregulation of Ang‐2.

**Figure EV1:**
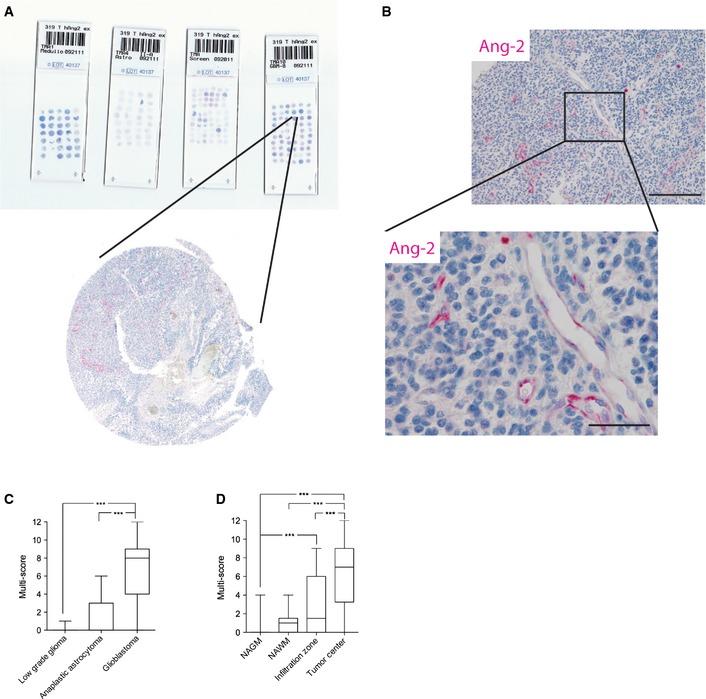
Application of tissue microarrays (TMAs) for the analysis of human glioma samples A, BBiopsies of 303 human gliomas (WHO grade II *, n *=* *16, WHO grade III,* n *=* *35, and WHO grade IV,* n *=* *252) were spotted on microscope slides (A) and processed for automated anti‐Ang‐2 immunohistochemistry (Ventana Benchmark platform) (B). Scale bar (B): 200 μm, inset: 50 μm.CAng‐2 expression was assessed by applying a semiquantitative scoring system (Harter *et al*, 2010).DSpatial expression of Ang‐2 in brain specimens from glioblastoma patients was scored in normal‐appearing gray matter (NAGM) (*n *=* *48), normal‐appearing white matter (NAWM) (*n *=* *18), infiltration zone (*n *=* *39), and tumor center (*n *=* *62). Data information: In (C, D), for statistical analysis, Kruskal–Wallis test (followed by Dunn's post‐test) was applied. ***P <* *0.005. Whisker Box plots displaying median, 25–75^th^ percentile, upper and lower quartile.

Next, we screened glioma biopsies for Ang‐2 protein expression. As vascular densities significantly advanced from diffuse low‐grade to high‐grade gliomas (WHO grades II‐IV), we analyzed the course of Ang‐2 expression among the different WHO grades [low‐grade diffuse glioma (*N* = 14), anaplastic astrocytoma (*N* = 12), glioblastoma (*N* = 11)] compared to healthy controls (*N* = 3). The number of Ang‐2‐positive vessels significantly increased among WHO grades as indicated in Fig 1D. The highest expression was observed in glioblastoma, whereas Ang‐2 expression was not detectable in the normal brain vasculature (Fig 1D). We then expanded the study by a large cohort of glioma specimens (*N* = 303, including controls) that were imprinted on tissue microarrays (TMAs) and processed to an automated anti‐Ang‐2 staining procedure using the Ventana Benchmark Platform (Fig EV1A and B). As evidenced by a multiscore analysis in Fig EV1C and D (see Materials and Methods for details), Ang‐2 expression in glioma vessels significantly increased from low‐grade diffuse glioma to glioblastoma (WHO grades II–IV), with highest expression in the tumor center.

We and others previously identified Ang‐2 mRNA as an early tumor marker that is specifically upregulated in endothelial cells of GBM (Stratmann *et al*, 1998; Zagzag *et al*, 1999). We now aimed to investigate the detailed spatial Ang‐2 protein expression in human GBM by means of double immunohistochemistry and high‐resolution confocal imaging (Fig 1E). Frozen, patient‐derived glioblastoma samples were co‐stained with the following antibody combinations: Ang‐2 and vWF, αSMA, or Iba1, respectively (Fig 1E). As shown in Fig 1E, Ang‐2 expression was restricted to endothelial cells and not detectable on tumor cells, pericytes, or microglia/macrophages. Sections of four different glioblastoma specimen compared to control brains are shown in Fig 1E.

Our TMA analyses (Fig EV1) confirmed that Ang‐2 expression is upregulated in the majority of glioma patients and is restricted to tumor neovessels (Fig 1E). Moreover, as shown in Fig 1F, high Ang‐2 expression levels in glioblastoma patients negatively correlated with survival, further suggesting that inference with angiopoietin/Tie signaling may be of therapeutic value. Collectively, our data generated from a comprehensive study group validate Ang‐2 as a prognostic marker and a potential therapeutic target in glioma.

### Ang‐2 gain of function in endothelial cells leads to an immature vascular phenotype and excess infiltration of innate immune cells in experimental glioma

We next aimed to test the functional consequences of continuous Ang‐2 signaling in experimental glioblastoma and applied the previously established Ang‐2 double‐transgenic (Ang‐2 DT) mouse model (Reiss *et al*, 2007). In this model, Ang‐2 expression is specifically targeted in endothelial cells by using the Tet^OFF^ system and a Tie1 promoter (Reiss *et al*, 2007; Coffelt *et al*, 2010; Scholz *et al*, 2011).

**Figure 2:**
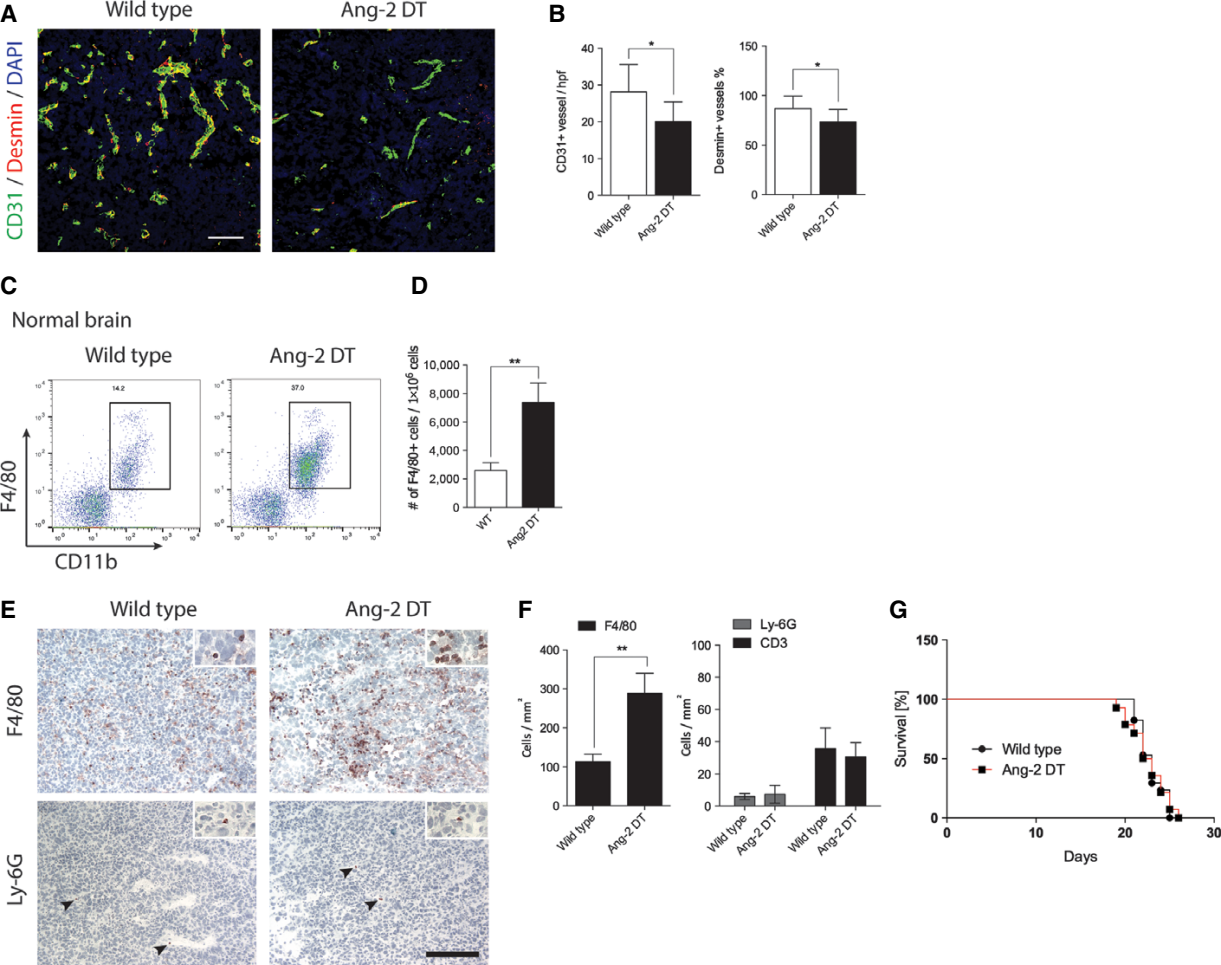
Endothelial Ang‐2 expression reduced pericyte coverage and increased macrophage infiltration in experimental glioblastoma GL261 cells were intracerebrally transplanted in wild‐type or Ang‐2 gain‐of‐function mice (Ang‐2 DT).
A, BBrain tumor sections were stained with antibodies against CD31 and desmin (A), and microvessel densities (MVD) and pericyte coverage were determined (WT *n *=* *14; Ang‐2 DT *n *=* *6) (B). Scale bar: 100 μm.C, D
FACS analysis of infiltrating macrophages in Ang‐2 DT normal brain (w/o tumor) compared to WT is shown (C, dot plot; D, quantification) (WT *n *=* *5; Ang‐2 DT *n *=* *3).E, F(E) Immunohistochemistry staining for monocytes/macrophages (F4/80) and neutrophils (Ly6G; arrowheads) in brain tumors derived from WT or Ang‐2‐overexpressing mice. (F) Quantitative analysis of (E). (WT *n *=* *14; Ang‐2 DT *n *=* *10). Scale bar: 100 μm.GKaplan–Meier survival curve of GL261 tumor‐bearing WT and Ang‐2 DT mice (WT *n *=* *17; Ang‐2 DT *n *=* *14).Data information: Statistical analyses were performed using an unpaired Student's *t*‐test (B, F), one‐way ANOVA (Tukey post‐test) (D), log‐rank and Wilcoxon (G); *P <* *0.05, **P <* *0.01; data are mean ± SEM.

GL261 glioma cells were orthotopically transplanted into the brain of Ang‐2 transgenic mice and wild‐type littermate controls. As demonstrated in Fig 2A and B, transgenic overexpression of Ang‐2 in endothelial cells led to an immature vascular phenotype of GL261 gliomas as evidenced by reduced pericyte coverage and fewer numbers of microvessels. An immature vascular phenotype in brain tumors of Ang‐2 transgenic mice most likely renders the possibility of vessel instability and permeability that facilitates the infiltration of innate immune cells. We previously showed that endothelial expression of Ang‐2 supports the recruitment of myeloid cells in peripheral organs that is largely enhanced under pathological conditions such as cancer and inflammation (Coffelt *et al*, 2010; Scholz *et al*, 2011). Consequently, we sought to analyze the recruitment of mononuclear cells in GL261 brain tumors upon endothelial‐specific expression of Ang‐2, and compare those findings to human glioblastoma.

As demonstrated by multicolor fluorescence‐activated cells sorting (FACS), Ang‐2 transgene expression led to significantly increased numbers of CD45^+^/CD11b^+^/F4/80^+^ inflammatory cells in the normal brain (Fig 2C and D). However, as expected, levels of inflammatory cells were considerably higher in GL261/Ang‐2 DT gliomas (Fig 2E and F). The infiltrates were specifically comprised of F4/80^+^ macrophages as determined by immunofluorescence analysis (Fig 2E and F). Although the tumor vasculature of Ang‐2 transgenic mice is considered immature, extravasation of cells from the blood appears to be selective and not random, as we have previously demonstrated by intravital microscopy in the vasculature of Ang‐2 DT mice (Scholz *et al*, 2011). Consequently, and as previously reported (Scholz *et al*, 2011), CD3^+^ lymphocytes and Ly6G^+^ neutrophil granulocytes were not effectively recruited into brain tumors of Ang‐2 DT mice (compared to wild‐type controls; Fig 2E and F). However, vascular changes and inflammatory cell recruitment did not influence the overall survival of Ang‐2 transgenic mice, most likely due to regional differences in the tumor microenvironment or oversaturation with transgenic Ang‐2 (Fig 2G).

**Figure 3:**
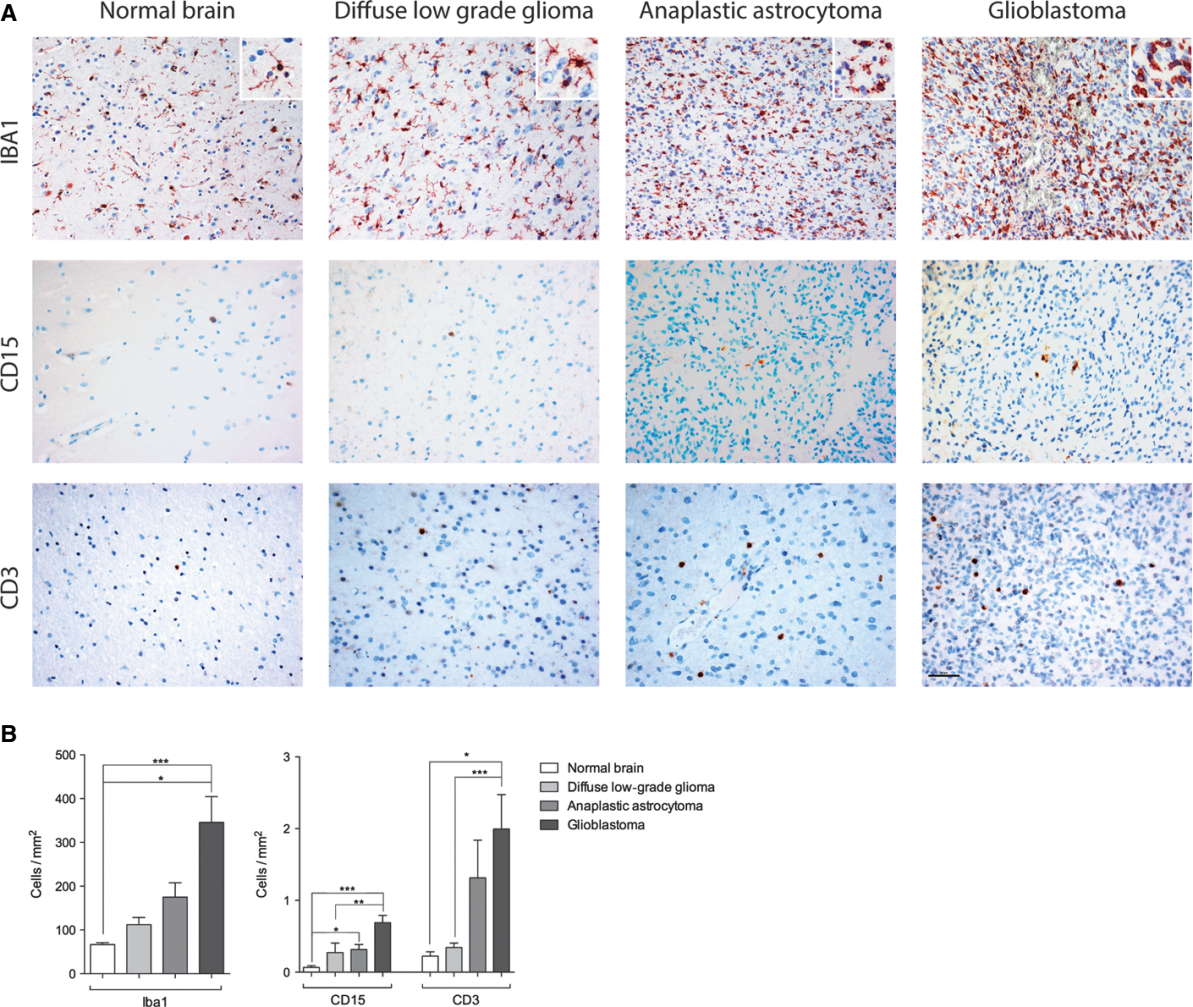
Infiltrating leukocyte subsets in human glioma A, BParaffin sections of healthy human brain (*n *=* *3), low‐grade glioma (*n *=* *12), anaplastic astrocytoma (*n *=* *7), and glioblastoma (*n *=* *9) were stained for IBA1 (macrophages/microglia), CD15 (granulocytes), and CD3 (T cells) (A). Quantification of leukocyte subsets in human glioma specimens (B). For statistical analysis, Kruskal–Wallis (Dunn's post‐test) test was applied. *P < 0.05, **P < 0.01, ***P < 0.005; data are mean ± SEM. Scale bar: 50 μm.

Tumor‐associated macrophages (TAMs) are known to interfere with angiogenic properties of tumors. In order to correlate our findings in the animal model with human neoplastic diseases, we investigated monocyte/macrophage infiltration in human glioma. WHO grade II‐IV glioma biopsies were processed for Iba1 immunohistochemistry (Fig 3A). In normal brain, macrophages/microglia are readily detectable but mostly inactive as judged by morphology criteria (ramified microglia with multiple processes; Fig 3A and insets therein). Macrophage/microglia numbers significantly increased with grade of malignancy concurrent with a change in mode of activation (ramified to ameboid phenotype, Fig 3A and insets therein). Interestingly, both Ang‐2 expression and the number of Iba1‐positive macrophages/microglia significantly increased with the grade of malignancy with highest expression in GBM (Fig 3A and B; compare to Fig 1D).

Similar to our findings in the animal model, Ang‐2 expression appears to be associated with the infiltration of monocytes/macrophages, whereas neutrophilic granulocytes (CD15) and T lymphocytes (CD3) were present in low amounts in the human glioma samples (Fig 3A and B). The number of infiltrating T cells increased in glioblastoma, but numbers were still low when compared to Iba1^+^ macrophages (Fig 3A and B). Collectively, our findings suggest that endothelial cell‐derived Ang‐2 is crucial for the recruitment of macrophages in mouse and human glioma and therefore may have an impact on the resistance to anti‐angiogenic therapy.

### Anti‐angiogenic therapy in glioblastoma: targeting Ang‐2 and VEGF signaling pathways

Increased numbers of myeloid cells in cancer are associated with dismal prognosis (Biswas *et al*, 2013) and are implicated in mediating therapy resistance in preclinical models (Shojaei *et al*, 2007; Chung *et al*, 2013). At present, FDA‐approved anti‐angiogenic therapy in GBM is restricted to the targeting of VEGF (Cohen *et al*, 2009). Given the presence of more numerous macrophages upon Ang‐2 expression in intracerebral tumors and the importance of Ang‐2 for vessel growth, we hypothesized that targeting the angiopoietin/Tie2 system may be beneficial for GBM patients.

We previously identified Ang‐2 as a regulator of myeloid cell infiltration in settings of inflammation (Scholz *et al*, 2011) and subcutaneous tumors (Coffelt *et al*, 2010). In those models, endothelial cell‐derived Ang‐2 promoted the recruitment of M2‐polarized, pro‐angiogenic macrophages (Coffelt *et al*, 2010; Scholz *et al*, 2011). Targeted inhibition of Ang‐2 signaling has been considered in preclinical tumor models but not particularly in GBM (Oliner *et al*, 2004; Brown *et al*, 2010; Hashizume *et al*, 2010; Koh *et al*, 2010; Mazzieri *et al*, 2011; Kienast *et al*, 2013; Thomas *et al*, 2013; Rigamonti *et al*, 2014).

To investigate whether Ang‐2 blockade is sufficient to prevent the influx of innate immune cells in experimental glioma, GL261 and GL261‐luc tumor cells were surgically transplanted intracranially into C57BL/6 mice and survival was assessed as a primary endpoint (see Materials and Methods for termination criteria). Untreated mice typically developed symptoms 21 days after implantation. We allowed tumors to establish for 5 days post‐surgical implantation before anti‐angiogenic treatment with AMG386 (Trebananib) (Oliner *et al*, 2004; Coxon *et al*, 2010), a peptibody that neutralizes Ang‐1 (IC50 = 6.2 nM) and, to a higher extent, Ang‐2 (IC50 = 0.029 nM), was initiated.

**Figure EV2:**
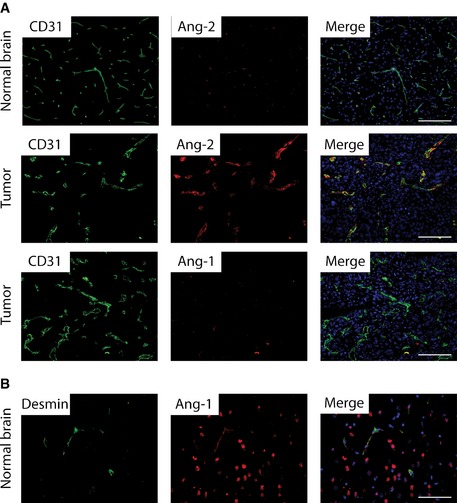
Angiopoietin expression in mouse brain and GL261 tumors A, BAng‐2 and Ang‐1 (red) immunofluorescence co‐staining with anti‐CD31 (green) was assessed in GL261 brain tumors (A). Ang‐2 is not detectable in normal brain (A). Ang‐1 (red) and desmin (green) double‐immunofluorescence staining in normal mouse brain (B). Scale bars: 100 μm.

**Figure 4:**
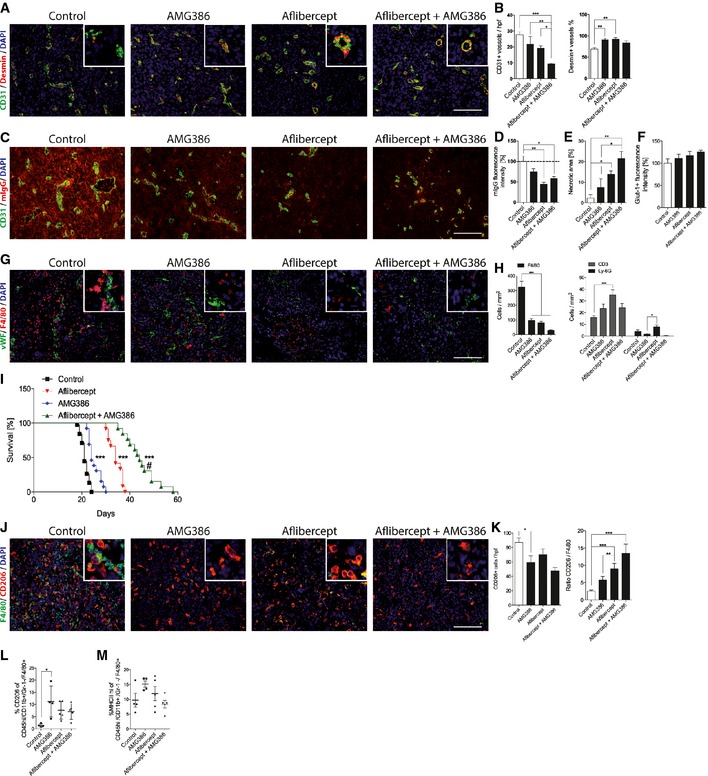
Dual anti‐Ang‐2 and anti‐VEGF therapy acts synergistic on vascular normalization and macrophage infiltration in experimental GBM A, BImmunofluorescence staining of CD31 and desmin in GL261 glioblastoma after single and dual treatment with anti‐Ang‐2 (AMG386) and anti‐VEGF (aflibercept) (A). Corresponding quantitative analysis of microvessel densities and pericyte numbers (B; control *n *=* *24; AMG386 *n *=* *10; aflibercept *n *=* *12; AMG386 +  aflibercept *n *=* *13).C, DStaining for mouse IgG (red) as a surrogate parameter for vascular leakage and CD31 (green) is shown in GL261 glioblastoma sections following different anti‐angiogenic treatments (C) with quantification in (D); *n *=* *3.EQuantification of necrotic areas in GL261 glioblastoma sections after anti‐angiogenic treatment is shown as percent of whole tumor area (control, aflibercept; AMG386 + aflibercept *n *=* *4; AMG386 *n *=* *3).FAnalysis of Glut1 immunoreactivity in GL261 glioblastoma sections (*n *=* *3).G, HDouble‐immunofluorescence stainings of macrophages (F4/80) and tumor vessels (vWF) in mouse GBM after treatment with AMG386, aflibercept, or the combination of both are shown in (G). Quantitative analysis of tumor‐infiltrating leukocytes (F4/80^+^, CD3^+^, Ly‐6G^+^) following anti‐angiogenic treatment is displayed in (H). (Control *n *=* *28; AMG386 *n *=* *12; aflibercept *n *=* *11; AMG386 +  aflibercept *n *=* *12).IKaplan–Meier survival analysis of GL261 tumors grown in C57BL/6 mice following anti‐angiogenic treatment (control *n *=* *38; AMG386 *n *=* *13; aflibercept *n *=* *12; AMG386 +  aflibercept *n *=* *13).J, KDouble‐immunofluorescence stainings with anti‐F4/80 and anti‐CD206 in brain tumor sections of mice treated with anti‐Ang‐2 (AMG386), anti‐VEGF (aflibercept), or the combination of both are shown in (J). Corresponding quantitative analyses of tumor‐infiltrating F4/80^+^ cells and CD206^+^ cells, and the ratio of CD206^+^ versus F4/80^+^ upon anti‐angiogenic therapy is displayed in (K) (control *n *=* *21; AMG386 *n *=* *13; aflibercept *n *=* *9; AMG386 +  aflibercept *n *=* *4).L, MFlow cytometry of tumor‐infiltrating macrophages following enzymatic dissociation of mouse GL261 brain tumors plus/minus anti‐angiogenic therapy. Percent of CD206^+^ (L) and MHC class II^HI^ cells (M) among CD45^+^ CD11b^+^ GR1^−^F4/80^+^ cells is displayed (control *n *=* *4; AMG386 *n *=* *4; aflibercept *n *=* *5; AMG386 +  aflibercept *n *=* *5).Data information: Statistical analyses were performed using one‐way ANOVA and Tukey's multiple comparison except for log‐rank and Wilcoxon (I). *P < 0.05, **P < 0.01, ***P < 0.005, ^#^ *P* < 0.005 of aflibercept + AMG386 versus aflibercept; data are mean ± SEM (A–K), mean ± SD (L, M). Scale bars (A, C, G and J): 100 μm.

Ang‐2 is known to be highly expressed early during tumor progression (Fig EV2) (Stratmann *et al*, 1998; Holash *et al*, 1999), whereas Ang‐1 levels largely remain unaltered. Therefore, we assume that AMG386 predominantly targets Ang‐2 in our GBM model. While anti‐Ang‐2 treatment had minimal effects on tumor microvessel densities, coverage with desmin^+^ pericytes was increased (Fig 4A and B). This observation is indicative for vessel normalization upon anti‐Ang‐2 therapy. In addition, Ang‐2 blockade with AMG386 led to reduced vascular permeability while necroses and hypoxia increased (Fig 4C–F). Necrotic areas were similar in Ang‐2 gain of function compared to wild‐type mice (data not shown). Furthermore, AMG386 significantly reduced the infiltration of F4/80^+^ macrophages (Fig 4G and H). At the same time, CD3^+^ T lymphocyte numbers were slightly increased (Fig 4H). Importantly, upon AMG386 treatment, the overall survival of GL261‐bearing mice significantly improved (Fig 4I, median survival 24 days).

Next, we investigated the consequences of VEGF inhibition in the GL261 and GL261‐luc glioma models. Mice that had undergone surgery were treated from day 5 onwards with solvent or aflibercept (Zaltrap^®^, VEGF‐trap). As shown in Fig 4A and B, microvessel densities of VEGF‐trap‐treated gliomas were significantly reduced, while pericyte numbers significantly increased compared to control tumors. Moreover, anti‐VEGF therapy led to a significant reduction of vascular permeability while necrotic and hypoxic areas increased (Fig 4C–F).

The examination of innate immune cell infiltration in the aflibercept treatment group revealed a significant decline in the number of F4/80^+^ macrophages (Fig 4G and H). Additionally, CD3^+^ T lymphocyte numbers were significantly increased upon aflibercept administration (Fig 4H). As a result, we observed a shift in the balance of T cells and macrophages (Fig 4H). While control mice developed neurological symptoms after 21 days of tumor cell inoculation (see Materials and Methods), survival of aflibercept‐treated mice was significantly extended (Fig 4I, median survival 34 days). Although the infiltration of macrophages was impeded and survival was extended, tumors were not eradicated completely. Likewise, GBM patients typically are not cured but rather develop escape mechanisms that necessitate the exploration of new therapeutic avenues. Consequently, we considered the dual targeting of VEGF and angiopoietin pathways for treatment of experimental glioma as described below.

**Figure EV3:**
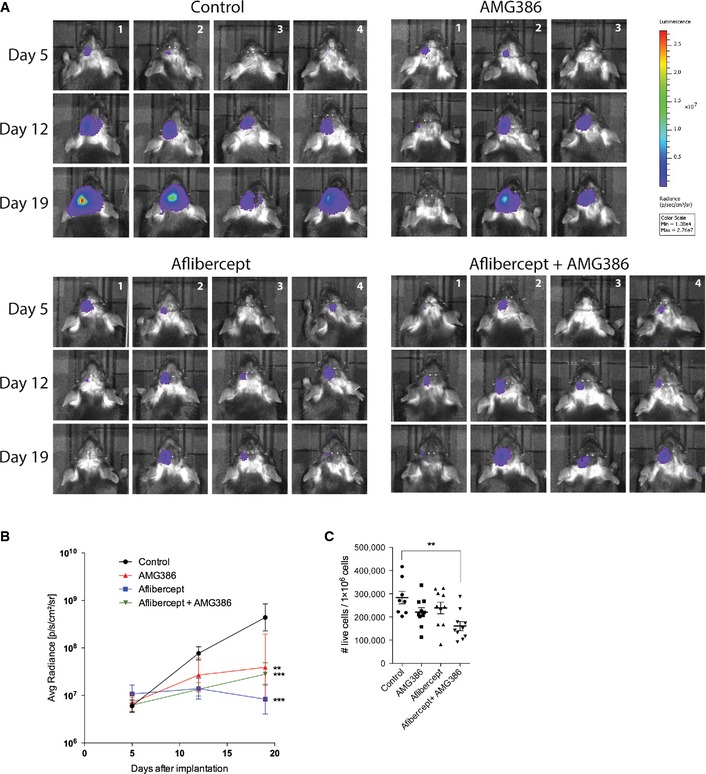
Noninvasive imaging of GL261 tumor growth following anti‐angiogenic therapy A, BBioluminescence imaging of GL261‐luc glioma cells on day 5, 12, and 19 after surgical implantation following AMG386, aflibercept, and combination treatment (A), and corresponding quantitative analysis (B). (Control *n *=* *4; AMG386 *n *=* *4; aflibercept *n *=* *3; AMG386 +  aflibercept *n *=* *4.)CFlow cytometric analysis of DAPI‐negative live cells in mouse Gl261 brain tumors following anti‐angiogenic therapy (control *n *=* *4; AMG386 *n *=* *4; aflibercept *n *=* *5; AMG386 +  aflibercept *n *=* *5). Data information: One‐way (C) and two‐way (B) ANOVA followed by Tukey post‐test were performed for statistical analysis, **P <* *0.01, ***P <* *0.005. Data are mean ± SEM (B), mean ± SD (C).

Despite beneficial outcome in preclinical tumor and metastasis models (Brown *et al*, 2010; Coxon *et al*, 2010; Hashizume *et al*, 2010; Koh *et al*, 2010; Kienast *et al*, 2013; Rigamonti *et al*, 2014), this is to our knowledge the first study that emphasized dual targeting of Ang‐2 and VEGF in GBM. As monotherapy targeting VEGF and Ang‐2, respectively, displayed highly reminiscent tumor phenotypes in the preclinical tumor model, we asked whether combination therapy of AMG386 and aflibercept could be beneficial with regard to overall survival of glioma‐bearing mice. Importantly, the effect was synergistic as indicated by the Kaplan–Meier curve (Fig 4I). Similarly, bioluminescence imaging, carried out over a time period of 3 weeks from the onset of therapy (not to be equated with therapy endpoint), already revealed a significant difference between control and treatment groups (Fig EV3). Moreover, overall survival (the primary endpoint) of mice treated with a combination of AMG386 and aflibercept was significantly increased, thus greatly exceeding the survival benefit reached in the single treatment groups (AMG386 plus aflibercept versus aflibercept alone, *P* < 0.005, Fig 4I, median survival 44 days). This is also supported by decreased numbers of live cells (determined by FACS) in the treatment groups, particularly upon dual targeting of Ang‐2 and VEGF (Fig EV3C). In line with the reported increase of necroses and hypoxia (Fig 4E and F), our results suggest that tumor burden is decreased following combination therapy. Subsequent immunohistological analyses revealed a normalized vasculature with fewer microvessels that were largely covered with perivascular mural cells (Fig 4A and B), accompanied by reduced vascular permeability (Fig 4C and D) and increased areas of necroses/hypoxia (Fig 4E and F). In addition, the presence of myeloid cells was significantly eradicated upon combination therapy while CD3^+^ T lymphocyte numbers increased to some extent (Fig 4G and H). The frequency of Ly‐6G granulocytes was very low in all different treatment groups (Fig 4H).

**Figure EV4:**
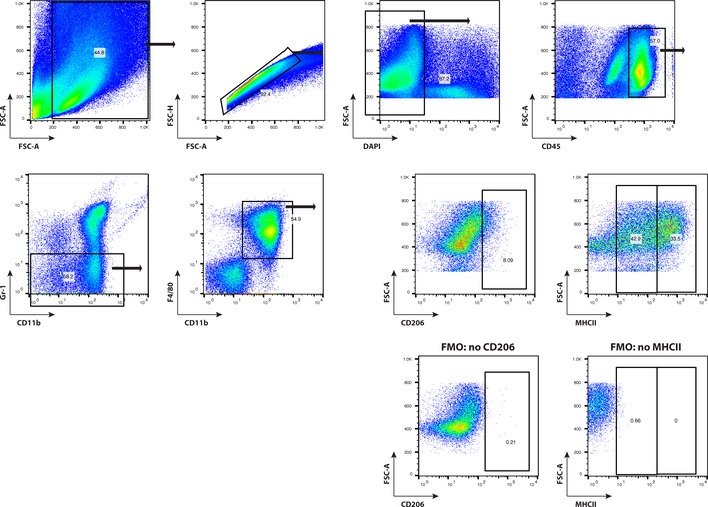
Flow cytometry of brain tumor‐infiltrating macrophages Gating strategy to identify brain tumor‐infiltrating macrophages is displayed. M2‐polarized, pro‐angiogenic macrophages are identified as CD45^Hi^ CD11b^+^Gr‐1^−^F4/80^+^ CD206^+^. M1‐polarized macrophages are identified by co‐expression of MHC class II (MHCII^H^ ^i^) on CD45^Hi^ CD11b^+^Gr‐1^−^F4/80^+^ TAMs.

We next aimed to investigate macrophage subpopulations in more detail. Macrophages are a heterogeneous population of innate immune cells that can be further subdivided in classically activated macrophages (M1‐polarized) and alternatively activated macrophages (M2‐polarized) depending on the cytokine milieu (Biswas *et al*, 2013). In the majority of studies, tumor‐associated macrophages were identified as M2‐polarized cells that exhibit pro‐angiogenic and immunosuppressive functions that are associated with poor prognosis (Biswas *et al*, 2013). To analyze whether anti‐angiogenic therapy affected a specific subpopulation of macrophages in mouse GBM, we stained AMG386‐, aflibercept‐treated, and control tumors for CD206 (macrophage mannose receptor/MMR) that is considered a marker for M2‐polarized macrophages (Fig 4J). While anti‐Ang‐2 and anti‐VEGF therapy led to significantly reduced numbers of F4/80^+^ macrophages (Fig 4G and H), cells c o‐expressing the M2‐polarization marker CD206 were less effectively depleted as evidenced by immunofluorescence (Fig 4J and K) as well as flow cytometry analyses (Fig 4L; (CD45^Hi^/CD11b^+^/Gr‐1^−^/F4/80^+^/CD206^+^; see Fig EV4 and Materials and Methods for gating strategy). Of note, and in line with previous reports, a subpopulation of CD206^+^/CD11c^−^ M2‐like macrophages expressed the Tie2 receptor tyrosine kinase (data not shown) that has been shown to be present on intratumoral pro‐angiogenic macrophages and to critically promote tumor growth (Venneri *et al*, 2007; Mazzieri *et al*, 2011). Further flow cytometry analyses of GL261 tumors revealed that M1‐polarized TAMs, defined by their expression of major histocompatibility complex class II (CD45^Hi^/CD11b^+^/Gr‐1^−^/F4/80^+^/MHCII^Hi^/CD206^−^; Fig EV4 for gating strategy), are not affected by anti‐angiogenic therapy (Fig 4M). Taken together, our findings imply that additional therapeutic strategies including the targeting of M2‐polarized macrophages may be required to successfully treat GBM.

Overall, we provide evidence that Ang‐2 inhibition is sufficient to block the entry of F4/80^+^ macrophages and convert tumor vessels toward a more stable vascular phenotype (vascular normalization). As such, Ang‐2 provides a potential clinical target for the treatment of glioblastoma.

### Therapy‐induced plasticity of the GBM microenvironment reveals novel targets in human GBM

In order to investigate whether observations made in the preclinical glioblastoma model are of relevance for human GBM, we analyzed patient‐derived tumor samples before and after therapy. GBM biopsies were divided in three different groups depending on the therapy received: (i) S, treatment‐naive GBM (post‐surgery, pre‐radiochemotherapy, pre‐bevacizumab, (ii) S/CTx/RTx (post‐surgery, post‐radiochemotherapy, pre‐bevacizumab), and (iii) S/CTx/RTx/Bev (post‐surgery/post‐radiochemotherapy/post‐bevacizumab). See Appendix Table S1 for detailed information on patients enrolled in this study.

**Figure 5:**
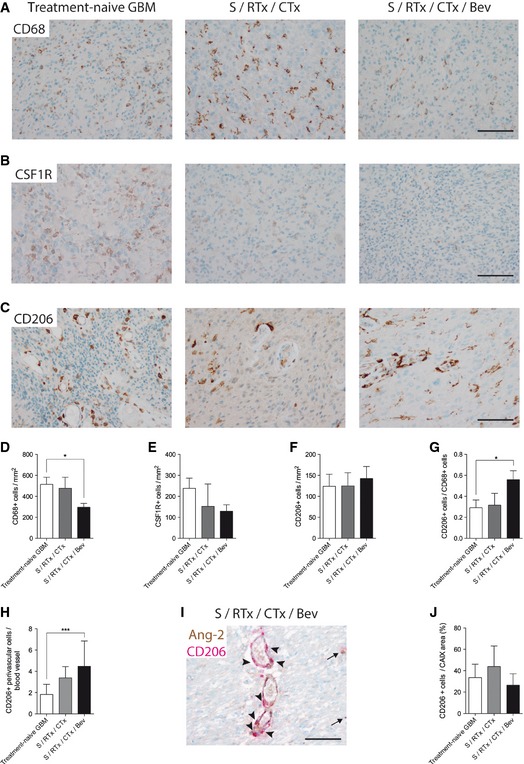
Anti‐VEGF therapy led to decreased infiltration of CD68^+^ macrophages in human GBM A–GAnti‐CD68 (A), anti‐CSFR1 (B), and anti‐CD206 (C) immunohistochemistry of patient samples derived from treatment‐naive GBM (*n *=* *24), post‐radiochemotherapy (S/RTx/CTx) (*n *=* *7), and post‐radiochemotherapy + bevacizumab (S/RTx/CTx/Bev) (*n *=* *29) therapy are shown. Corresponding quantification of tumor‐infiltrating cells is displayed (anti‐CD68, D), (anti‐CSF1R, E), and (anti‐CD206, F). Ratio of CD206^+^ versus CD68^+^ cells (G).HAmount of CD206^+^ perivascular cells per blood vessel.IDouble staining of Ang‐2 (brown) and CD206 (red) of a S/RTx/CTx/Bev patient, arrowheads indicating perivascular CD206^+^ cells around Ang‐2^+^ vessels. Arrows pointing on parenchymal CD206^+^ cells.J
CD206^+^ cells in relation to the percentage of hypoxic (CAIX positive) tumor area.Data information: Statistical analysis: Kruskal–Wallis (Dunn's post‐test). *P <* *0.05, ***P <* *0.005; data are mean ± SEM. Scale bars (A–C, I): 100 μm.

Similar to VEGF blockade in our rodent GBM models, CD68^+^ macrophages were significantly reduced in the S/CTx/RTx/Bev group (Fig 5A and D) but not after S/CTx/RTx therapy alone (Fig 5A and D). Next, we examined expression levels of CSF1R. CSF1R is important for the differentiation and survival of macrophages and therefore is under investigation as a target in cancer therapy (Pyonteck *et al*, 2013; Ries *et al*, 2014). In line with our CD68 expression data, CSF1R^+^ macrophages were slightly reduced in S/CTx/RTx‐ and S/CTx/RTx/Bev‐treated GBM compared to treatment‐naive GBM (Fig 5B and E).

We next aimed to identify further subtypes of infiltrating macrophages and therefore processed human GBM biopsies for CD206 immunohistochemistry (Fig 5C). While total CD206^+^ cell numbers did not decrease in the three different therapy groups, the ratio of CD206^+^ versus CD68^+^ cells significantly increased in the S/CTx/RTx/Bev group (Fig 5F and G). Moreover, the spatial distribution of CD206^+^ cells in the tumor microenvironment changed in a treatment‐dependent manner. Compared to treatment‐naive GBM, the number of perivascular CD206^+^ cells increased in S/CTx/RTx‐ and S/CTx/RTx/Bev‐treated GBM (Fig 5H and I). In contrast, the number of CD206^+^ cells that were associated with perinecrotic areas was not affected by treatment (Fig 5J). Therefore, similar to our findings in the GL261 model, we identified that remaining macrophage populations in bevacizumab‐treated GBM patient biopsies were preferentially polarized toward the pro‐angiogenic M2 phenotype and may thus contribute to therapy resistance following treatment with bevacizumab.

**Figure 6:**
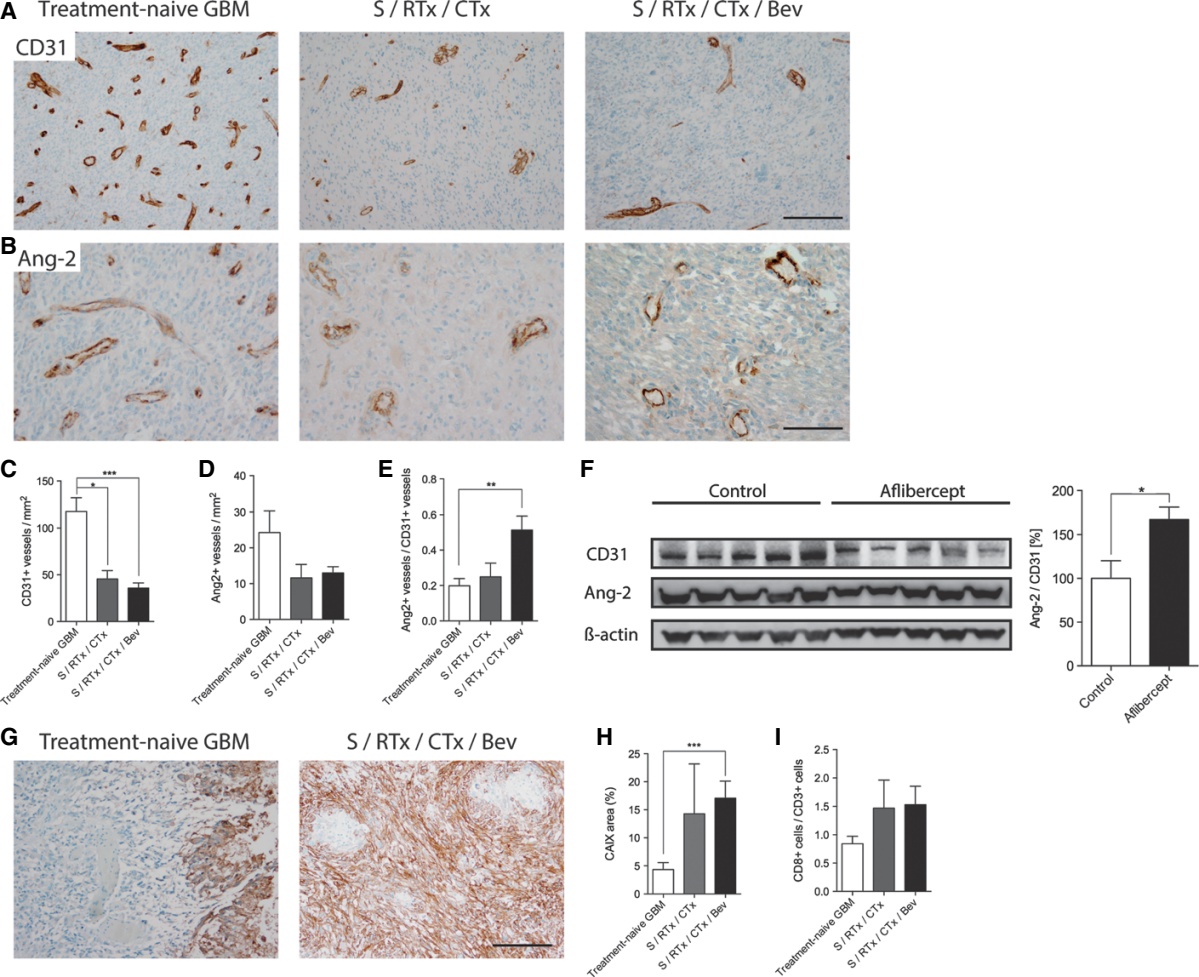
Bevacizumab therapy leads to reduced vessel density and increased Ang‐2 expression A–EImmunohistochemistry stainings with antibodies directed against CD31 (A) and Ang‐2 (B) in treatment‐naive GBM, post‐radiochemotherapy (S/RTx/CTx), and post‐radiochemotherapy + bevacizumab (S/RTx/CTx/Bev) therapy are displayed. Quantitative analyses of microvessel densities and Ang‐2^+^ vessels are shown in (C) and (D). The ratio of Ang‐2^+^ versus CD31^+^ vessels was determined in (E). Scale bars: 200 μm (A), 100 μm (B).FWestern Blot and corresponding quantification of CD31 and Ang‐2 in control and aflibercept‐treated mice (*n* = 5 each).G–I
CAIX staining of treatment‐naive GBM and S/RTx/CTx/Bev patients. Analyses of hypoxic (CAIX ^+^) area with regard to the whole tumor in human GBM (H). Tumor‐infiltrating lymphocytes (CD8/CD3) in human patients (I). Scale bar: 100 μm.Data information: Kruskal–Wallis (C–E, H–I). *P <* *0.05, **P <* *0.01, ***P <* *0.005; treatment‐naive GBM *n *=* *26, S/RTx/CTx *n *=* *7, S/RTx/CTx/Bev *n *=* *29; *t*‐test (F); data are mean ± SEM.

Furthermore, while vessel densities significantly declined from treatment‐naive to bevacizumab‐treated GBM samples (120 CD31^+^ vessels/mm^2^ versus 40 CD31^+^ vessels/mm^2^, *P* < 0.005; Fig 6A and C), the vast majority of the remaining tumor vessels in the bevacizumab‐treated group showed strong Ang‐2 immunoreactivity (Fig 6B, D and E). In line with this finding, serum Ang‐2 levels remained unchanged in patients before and after bevacizumab therapy, despite a 66% decrease in the number of CD31^+^ endothelial cells, which are the cellular source of Ang‐2 in GBM. These findings are even further supported by Western blot results obtained in the mouse GBM model (Fig 6F) where Ang‐2 was strongly upregulated in gliomas of mice treated with aflibercept. Thus, the increased Ang‐2 protein levels in vessels that remained upon bevacizumab therapy further identify Ang‐2 as a potential contributor to therapy resistance/relapse following anti‐VEGF therapy in GBM, a concept that only recently arose from findings in models of pancreatic neuroendocrine tumors (Rigamonti *et al*, 2014), brain cancer metastasis (Avraham *et al*, 2014), and U87 xenografts (Burrell *et al*, 2014). Since Ang‐2 is regulated by oxygen levels, we also investigated the surrogate hypoxia markers carbonic anhydrase IX (CAIX) in human GBM (Fig 6G and H) and glucose transporter‐1 (GLUT1) in murine GBM (described in Fig 4F, see above), and compared those findings to the size of gross hemorrhagic and necrotic areas in treated versus treatment‐naive GBM. There was no evidence for gross hemorrhages in treatment‐naive and treated tumors (data not shown). However, and in line with previous observations (Koh *et al*, 2010), hypoxic and necrotic areas increased in treated tumors and were largest in the combination therapy (VEGF and Ang‐2 inhibition) group as described above (Fig 4E and F). Thus, tumor hypoxia that is aggravated by chemotherapy and anti‐angiogenic therapy may further upregulate endothelial Ang‐2 in a subset of vessels and thus drive tumor progression via vascular destabilization, increased vascular permeability and therapy‐induced recruitment and/or reeducation of TAMs (Quail & Joyce, 2013).

Of interest, bevacizumab therapy also affected the ratio of CD8/CD3^+^ T lymphocytes that was highest in the S/RTx/CTx/Bev group (Fig 6I), suggesting that VEGF inhibition affects the intratumoral composition of T cells, as previously reported by others (Huang *et al*, 2012; Chung *et al*, 2013; Motz *et al*, 2014).

In summary, we observed a significant reduction of macrophages upon bevacizumab therapy. Importantly, CD206^+^ subsets did not decline, in fact their relative number (ratio CD206/CD68) increased, as well as their perivascular association (Fig 5F–H). Since perivascular CD206^+^ macrophages are associated with the M2‐phenotype that displays pro‐angiogenic and pro‐tumorigenic properties (Hughes *et al*, 2015), our findings argue for specific targeting of CD206^+^ macrophages following anti‐angiogenic therapy. Furthermore, despite microvascular densities significantly declined upon bevacizumab therapy, the relative expression of Ang‐2 in the remaining vessels increased, resulting in serum levels of Ang‐2 that were similar in pre‐ compared to post‐bevacizumab patients. Taken together, these findings argue strongly for the targeted therapy of Ang‐2 in conjunction with VEGF in glioblastoma patients, potentially followed by the targeting of CD206^+^ macrophages.

## Discussion

In this study, we identify the angiopoietin/Tie2 signaling pathway as a potential target for treatment‐naive as well as bevacizumab‐treated glioblastoma. We define a non‐neoplastic cell type, namely endothelial cells as the sole source of Ang‐2, and imply these cells in therapy resistance. While Ang‐2 is highly upregulated in treatment‐naive GBM, its expression is not intrinsically detectable in normal human brain, suggesting that Ang‐2 might be a suitable therapeutic target. In GBM biopsies derived from patients that received bevacizumab therapy following standard radiochemotherapy, vessel density decreased compared to the pre‐treatment biopsy, whereas Ang‐2 expression was increased in the remaining tumor vessels. Further, our study suggests a therapy‐induced plasticity of innate immune cells, resulting in less, but preferentially M2‐polarized macrophages accompanied by an increase in intratumoral T cells. Mechanistically, combined anti‐angiogenic therapy displayed a synergistic effect on both the inflammatory and vascular compartment in the murine syngeneic glioblastoma models GL261 and GL261‐luc. We therefore conclude that combining anti‐VEGF and anti‐Ang‐2 therapy in GBM patients might be superior to VEGF inhibition alone.

Anti‐angiogenic therapy using bevacizumab has been proven successful in a number of tumor entities such as recurrent colorectal cancer, renal cell carcinoma, and ovarian cancer (Carmeliet & Jain, 2011; Welti *et al*, 2013). However, several tumor types including glioblastoma are not well responsive or have been shown to develop escape mechanisms that lead to further tumor progression, despite anti‐angiogenic therapy (Batchelor *et al*, 2014). Therapy resistance of tumors has partly been attributed to the infiltration of TAMs that promote tumor growth by the secretion of pro‐angiogenic cytokines (Shojaei *et al*, 2007; Chung *et al*, 2013; De Palma & Lewis, 2013). Within the study presented here, we show that the combined inhibition of the two major angiogenic signaling pathways (VEGF/VEGFR and angiopoietin/Tie2) led to improved survival and the depletion of pro‐angiogenic macrophages. As such, targeting of Tie2 signaling in addition to blockade of VEGF may be of clinical relevance to overcome bevacizumab resistance in GBM patients.

The concept of combined targeting of VEGF and Ang‐2/Tie2 signaling has been considered previously. A number of preclinical studies clearly demonstrated a beneficial outcome in different tumor entities (Brown *et al*, 2010; Hashizume *et al*, 2010; Koh *et al*, 2010; Daly *et al*, 2013; Kienast *et al*, 2013; Rigamonti *et al*, 2014). Importantly, none of those studies involved the investigation of combined VEGF and Ang‐2 therapy in GBM, the most common and most aggressive malignant primary brain tumor in humans. Prognosis for GBM patients is dismal with a median overall survival of 15 months, despite recently optimized therapy that includes combined radiochemotherapy following surgical resection (Stupp *et al*, 2005). As such, the quest for new treatment options is perpetual. In the present study, we sought to explore anti‐VEGF and anti‐Ang‐2 therapy in orthotopic, syngeneic models of glioblastoma by intracranial transplantation of GL261 and GL261‐luc cells. The implementation of syngeneic glioma models allows to investigate the contribution of the host immune system for tumor progression. These preclinical studies are complemented by a unique cohort of biopsies derived from patients that received bevacizumab (S/RTx/CTx/Bev: post‐surgery, post‐radiochemotherapy, post‐bevacizumab). Samples denominated S/RTx/CTx consist of biopsies from the same patients after radiochemotherapy but before bevacizumab treatment, whereas samples denominated treatment‐naive comprise biopsies from the patients before radiochemotherapy and bevacizumab treatment. Importantly, these samples were derived from the same patients during the clinical course of the disease.

A major finding of our study can be attributed to a significant reduction of TAMs upon anti‐angiogenic therapy, achieved by either treatment (AMG386 or aflibercept) alone or in combining both. In the GL261 model, blockade of Ang‐2 and/or VEGF led to a dramatic decrease of F4/80^+^ macrophages that coincided with increased survival. Our results are in line with previous own findings demonstrating that continuous expression of Ang‐2 in the vasculature of transgenic mice led to an accumulation of pro‐angiogenic macrophages in subcutaneous lung carcinomas (Coffelt *et al*, 2010) or in settings of inflammation (Scholz *et al*, 2011). Vice versa, Ang‐2 deficiency impaired the recruitment of myeloid cells to sites of inflammation (Fiedler *et al*, 2006). Based on these findings, we hypothesized that targeting of Ang‐2/Tie signaling would improve overall survival in the preclinical GBM model. Indeed, macrophage depletion correlated with increased survival. Although the blockade of angiopoietin/Tie2 signaling has previously be shown to prevent the recruitment of CD11b^+^ myeloid cells in a model of airway inflammation (Tabruyn *et al*, 2010) and in colon cancer (Huang *et al*, 2011), this study extends these observations to a mouse model of brain cancer. In addition, and coinciding with the elimination of macrophages, blocking of Ang‐2 led to “vessel normalization,” as evidenced by pruning of the chaotic vasculature, decreased vascular permeability and increased pericyte coverage (Jain, 2005). It has been shown that VEGFR2 blockade can temporarily normalize tumor vessels via the expression of Ang‐1 (Winkler *et al*, 2004). As a consequence, transient stabilization of vessels and improved oxygen delivery to hypoxic zones is achieved following VEGF neutralization. However, the combined blockade of VEGF and angiopoietin signaling in our preclinical glioma model reduced vessel numbers and increased necroses/hypoxia even further as compared to the single treatment groups alone.

Although macrophage numbers dramatically decreased, some residual tumor‐associated macrophages remained, in both the single treatment and the combination treatment groups. The remaining macrophages preferentially displayed an alternatively activated, M2‐skewed phenotype as indicated by the expression of the mannose receptor (MMR/CD206). Survival in the AMG386/aflibercept combination treatment group was significantly improved although tumors were not eradicated completely. Of note, our findings in the preclinical GBM model are in line with the data derived from the different human GBM therapy groups. Here, we observed a therapy‐induced overall reduction of TAMs combined with a relative increase of CD206^+^ TAMs that further displayed a preferential perivascular localization. Interestingly, our observations are in line with recent findings suggesting that a subpopulation of TAMs, defined as CD206^+^/TIE2^Hi^/CXCR4^Hi^, accumulate around blood vessels after chemotherapy in breast cancer, where they promote tumor revascularization and relapse, partly by release of VEGF (Hughes *et al*, 2015). Thus, our findings suggest that subsequent targeting of M2‐polarized macrophages might potentially be useful to overcome therapy resistance in glioblastoma. Along this line, we have previously shown that the functional targeting of bone marrow‐derived monocytes using a mutant, signal‐transduction incompetent VEGFR‐1 significantly slows tumor growth in the same GL261 glioma model as applied in the current study (Kerber *et al*, 2008). Furthermore, recent preclinical studies as well as a clinical trial in diffuse‐type giant cell tumor patients provided evidence that the functional blockade of CSF1R impairs tumor‐promoting functions either by decreasing total TAM number or, alternatively, by reeducating TAMs (Pyonteck *et al*, 2013; Ries *et al*, 2014). As such, in a proneural GBM model, CSF1R monotherapy by use of the blood–brain barrier permeable inhibitor BLZ945 displayed a decrease in M2‐polarized macrophages, whereas the total number of TAMs was unaltered (Pyonteck *et al*, 2013). In line with our observations, those studies imply that the combined targeting of angiogenesis and innate immune cells may be advantageous (Quail & Joyce, 2013). Of note, anti‐CSF1R therapy appears to be dependent on the M1/M2 macrophage ratio within a given tumor (Ries *et al*, 2014). Since the CD206/CD68 ratio increases in bevacizumab‐treated glioblastomas (“M2‐rich”), one may envision the application of CSF1R inhibitors in GBMs that recur after bevacizumab therapy. Clearly, further studies are necessary to prove whether the combination of anti‐angiogenic drugs with functional TAM inhibitors will be beneficial for brain cancer therapy.

Nevertheless, TAM targeting alone may not be sufficient to completely eradicate tumor growth. A recent report identified modes of resistance toward anti‐angiogenic therapy mediated by T lymphocytes (Chung *et al*, 2013). While anti‐VEGF therapy in a preclinical cancer model eradicated myeloid‐derived suppressor cells (MDSCs), remaining T cells triggered the infiltration of additional myeloid cells through the IL‐17 pathway (Chung *et al*, 2013). Although TAMs were depleted initially, remaining T cells triggered the secretion of G‐CSF by stromal cells that subsequently promoted the recruitment of pro‐angiogenic myeloid cells (Chung *et al*, 2013). Of interest, in the bevacizumab‐treated GBM cohort (S/RTx/CTx/Bev), the CD8/CD3 ratio was elevated compared to treatment‐naive GBM, suggesting that VEGF blockade affects T‐cell composition within tumors, which is in line with previous observations (Huang *et al*, 2012; Chung *et al*, 2013; Motz *et al*, 2014). Overall, these findings imply that combinatorial targeting of multiple cell types within the tumor microenvironment and/or their signaling pathways may be mandatory to completely eradicate tumor growth.

In summary, our findings clearly support previous studies which suggest that therapy resistance in preclinical models is at least partially mediated by upregulation of Ang‐2 in the vasculature (Avraham *et al*, 2014; Burrell *et al*, 2014; Rigamonti *et al*, 2014). Our data in human GBM were highly recapitulated in the preclinical GBM model and strongly suggest the involvement of angiopoietin signaling in the remodeling of the tumor environment, potentially by supporting several key events that not only include angiogenesis and vascular destabilization but additionally imply the infiltration and/or reeducation of TAMs, combined with immunosuppression. Consequently, in the preclinical GBM model, combined inhibition of VEGF‐ and angiopoietin signaling delayed tumor growth and significantly increased survival. Multiple mechanisms including angiogenesis inhibition, increased vascular normalization, decreased vascular permeability, reduction of TAMs, along with a partial relief of immunosuppression by enhancing the number of intratumoral T cells may underlie these effects. Taken together, these findings strongly argue for clinical trials targeting the angiopoietin signaling pathway in GBM, in particular in conjunction with anti‐VEGF therapy.

## Materials and Methods

### Reagents

The following reagents were applied: anti‐human Ang‐2 antibody (MAB 0983) 1:100 from R&D Biosciences, Minneapolis, USA; anti‐human Ang‐2 1:200 (#PA5‐27297, Pierce Biotechnology, Rockford, USA); anti‐Iba1 antibody 1:100, anti‐human CD31 antibody (clone JC704) 1:200, anti‐human CD68 (clone PG‐M1) 1:500, anti‐human CD3 (A04052) 1:500, anti‐human CD8 (C8/144B) 1:100, anti‐human CD15 (clone C3D1) 1:50, anti‐human vWF 1:100, anti‐human Desmin (clone D33), all from DAKO, Glostrup, Denmark; anti‐CSF1R (clone SP211) 1:100, anti‐human CD206 (clone 5C11) 1:100 from Abcam (Cambridge, United Kingdom). Anti‐human carboanhydrase IX (CAIX) (#NB100‐417) 1:2,000 from Novus Biologicals (Littleton, CO, USA). Anti‐mouse CD31 (clone MEC 13.1) 1:100, anti‐mouse Ly‐6G (clone 1A8) 1:100, anti‐mouse CD3e (clone 145‐2C11) 1:100 all from BD Pharmingen; anti‐mouse F4/80 (clone A3‐1) 1:100 from Biozol; goat anti‐mouse CD206 (clone C068C2) 1:100 from R&D Systems; anti‐αSMA (clone 1A4) 1:200 from Sigma; anti‐mouse Glut1 (polyclonal, C‐Terminus) 1:100 from Merck Millipore; anti‐human Ang‐1 (Santa Cruz, sc‐6319); and anti‐mouse Ang‐2 (R&D Systems, AF7186). Anti‐mouse Ang‐2 (Abcam), anti‐mouse CD31, and anti‐β‐actin (both Santa Cruz) were applied for Western blot analyses. Secondary antibodies were purchased from Dianova Hamburg, Germany, and Roche Diagnostics, Mannheim, Germany. Vectastain ABC and peroxidase substrate kits from Vector Laboratories, Burlingame, USA, were applied. For immunofluorescence stainings, Alexa Fluor secondary antibodies from Molecular probes were applied. The following flow cytometry antibodies (anti‐mouse) were used: CD45 PercP Cy5.5 (clone 30‐F11, BD Biosciences), Gr‐1 APC‐Cy7 (clone RB6‐8C5, BD Biosciences), CD11b APC (clone M1/70, BD Biosciences), F4/80 FITC (clone BM8, eBioscence), CD206 PE‐Cy7 (clone C068C2, Biolegend), and MHC class II PE (clone M5/114.15.2, BD Biosciences).

### Transgenic animals

We applied the tetracycline‐controlled transcriptional activation system (Tet^OFF^) for the generation of Ang‐2Tet^OS^:Tie1tTA double‐transgenic (gain of function) mice as previously described (Reiss *et al*, 2007). Mice were fed with doxycycline (Dox)‐containing food pellets (100 mg/kg) from ssniff Spezialdiaeten GmbH, Soest, Germany. To allow transgene expression, Dox was removed 2 weeks prior to GL261 tumor cell implantation. Transgene and control animals of both sexes (backcrossed in the C57BL/6 background) were used at 8–12 weeks of age. For FACS analysis of brain‐infiltrating macrophages, mice of > 6 months of age were used. Wild‐type littermates that inherited one or no transgene served as experimental controls. The present study was performed in accordance with the German Legislation on the Protection of Animals and the Guide for the Care and Use of Laboratory Animals with permission of the Regierungspraesidium Darmstadt, Germany.

### Immunocompetent intracranial GBM model/anti‐angiogenic therapy

Ang‐2 double‐transgenic mice (Ang‐2Tet^OS^:Tie1tTA) and corresponding littermate controls were depleted of Dox 2 weeks prior to GL261 tumor cell inoculation (Reiss *et al*, 2007). For anti‐angiogenic therapy, female C57BL/6 mice (Harlan Laboratories, the Netherlands) were used at 8 weeks of age. Mice were anesthetized by intraperitoneal injection of ketamine/xylazine (100 and 10 mg/kg body weight) and inserted in a stereotactic device (Stolting). GL261 glioma cells (gift from M. Machein, Freiburg, Germany) were cultivated in DMEM ‐GlutaMAX‐I (Invitrogen) supplemented with 10% FCS. For bioluminescence imaging, GL261 cells stably transfected with firefly luciferase gene (GL261‐luc; gift from B. Becher, Zürich, Switzerland) were applied. 1 × 10^5^ GL261 or GL261‐luc cells in 2 μl PBS were injected in the mouse striatum using a Hamilton syringe equipped with a 30‐G needle. Coordinates relative to bregma were the following: 0.5 (anterior–posterior), 2 (mediolateral), and 3.5 (dorsoventral). The health status of tumor‐bearing mice was checked on a daily routine. Mice were sacrificed when neurological symptoms appeared (starting at 3 weeks after intracerebral implantation). Twenty percent loss of body weight or prolonged weight loss accompanied with reduced water/food uptake, or hunched back, rough coat, paresis, tremor, ataxia, lethargia were considered as criteria for termination according to the Society of laboratory animals (GV‐Solas; http://www.gv-solas.de). To assess overall survival of treated and untreated animals, Kaplan–Meier curves were generated. Following intracerebral implantation, GL261 tumor cells were allowed to establish for 5 days before anti‐angiogenic therapy using AMG386 (Ang‐1/Ang‐2 peptibody; Amgen, Thousand Oaks, USA) (Oliner *et al*, 2004) and aflibercept (VEGF‐trap) (Sanofi‐Aventis, Frankfurt, Germany) (Holash *et al*, 2002) was initiated. In contrast to bevacizumab, that only targets VEGF‐A, aflibercept targets also VEGF‐B and PlGF. As such, our analysis of patients receiving bevacizumab might not be readily comparable to mice treated with aflibercept. However, although PlGF has initially been implicated in therapy resistance to anti‐angiogenic therapy, its role (as well as the role of VEGF‐B) in tumor angiogenesis remains under discussion (Fischer *et al*, 2007; Bais *et al*, 2010). In fact, it has been recently suggested that PlGF is not involved in therapy resistance to bevacizumab in GBM patients (Schneider *et al*, 2015). We therefore assume that the phenotype observed upon aflibercept therapy mainly reflects VEGF‐A inhibition. AMG386 (Trebananib^®^) was injected subcutaneously twice a week at doses of 5.6 mg/kg as described previously (Coxon *et al*, 2010). Aflibercept (Zaltrap^®^) was administered subcutaneously twice a week at doses of 25 mg/kg body weight (Holash *et al*, 2002). Subcutaneous injections of PBS or sterile saline were applied in control animals. Therapy was continued until mice became symptomatic.

### *In vivo* bioluminescence imaging

GL261‐luc glioma‐bearing mice were injected intraperitoneally with VivoGlo^™^ Luciferin, Promega (1.5 mg/ml). Ten minutes later, mice were anaesthetized and processed for image analysis using a IVIS Lumina II charge‐coupled device (CCD) imaging system (Caliper, PerkinElmer) for 2 min. Tumor growth was monitored once a week starting on day 5 post‐implantation for 3 weeks. Imaging data were analyzed and quantified with the Living Image Software for IVIS^®^ Lumina II (PerkinElmer).

### Immunohistochemical and immunofluorescence analyses of mouse brain tumors

Ten‐micrometer frozen sections of GL261 tumors were air‐dried and fixed in 4% PFA for 10 min followed by an endogenous peroxidase blocking step (1.5% H_2_O_2_ in methanol) for additional 10 min. Non‐specific binding sites were blocked in 5% BSA/0.01% Triton X‐100 in PBS (30‐min incubation), followed 20% normal goat serum (NGS) in 0.01% Triton X‐100 for 60 min at room temperature (RT). The following primary rat anti‐mouse antibodies diluted in 10% NGS/PBS/0.01% Triton X‐100 were used: anti‐Ly‐6G, anti‐CD3, and anti‐F4/80. Biotin‐conjugated secondary antibodies were purchased from Invitrogen. Detection was performed using Vectastain ABC Kit and AEC kit (Vector) followed by counterstaining with Mayer′s hemalum solution (Merck). Tumor‐infiltrating cells in the entire tumor area were stereologically counted using a Zeiss microscope with Stereo Investigator 4.34 Software from MicroBrightField, Inc.

For double‐immunofluorescence stainings with markers against anti‐CD31/desmin, anti‐F4/80/CD206, anti‐CD31/mouse IgG, and CD31/CD206, slides were fixed in ice‐cold 95% EtOH for 5 min and acetone at RT for 1 min. Consecutive washing was carried out in PBSA solution (150 mM NaCl, 10 mM Na_2_HPO_4_, 10 mM KH_2_PO_4_, 1% BSA, and 0.1% Triton X‐100; pH 7.5). Primary antibodies were applied for 1 h at RT in antibody dilution buffer (0.5% BSA, 0.25% Triton X‐100 in PBS, pH 7.2), followed by incubation with Alexa Fluor‐labeled secondary antibodies (1 h at RT). Slides were post‐fixed in 4% PFA for 10 min, counterstained with DAPI, and embedded in Aqua PolyMount. Anti‐F4/80/vWF double immunofluorescence was carried out on 4% PFA‐fixed cryosections. After blocking of non‐specific binding sites with 5% BSA and 20% NGS, respectively, primary antibodies were incubated over night at 4°C in 10% NGS. Sections were then incubated with corresponding Alexa Fluor‐labeled secondary antibodies for 1 h at RT. Sections were counterstained with DAPI and embedded in Aqua PolyMount.

For mouse Ang‐1 and Ang‐2 immunofluorescence staining, cryosections were fixed in methanol: acetone (1:1) for 10 min at −20°C and air‐dried for 5 min before they were washed in PBS three times. The sections were then incubated in serum‐free blocking buffer (DAKO, X0909) for 10 min at RT, followed by an overnight incubation with primary antibodies in PBST at 4°C [anti‐human Ang‐1 (Santa Cruz), anti‐mouse Ang‐2 (R&D), anti‐human desmin, anti‐mouse CD31], and a subsequent 1 h incubation with corresponding secondary antibodies at RT (Alexa Fluor antibodies, Molecular Probes). The sections were then stained with DAPI and mounted. For histological analyses, five images of each tumor (*N* = 3–24) were acquired at 4–200× magnification using a Nikon ecplise 80i microscope. Microvessel densities, pericyte coverage, immune cell infiltration, permeability, hypoxia, and necroses were assessed using Nikon NIS Elements Software by determination of marker‐positive areas or fluorescence intensities.

### The cancer genome atlas (TCGA) data analyses

To determine gene expression of the Angpt/Tie system in gliomas, we analyzed data from the cancer genome atlas TCGA platform (https://tcga-data.nci.nih.gov/tcga/; accession date 09/23/2015). The TCGA glioblastoma cohort (TCGA GBM: GBM IV) comprised expression profiles of 553 GBM IV patients measured on Agilent 244K Custom Gene Expression G4502A‐07‐1 or G4502A‐07‐2 microarrays. The gene expression measurements were provided as log2 ratios of tumor compared to a normal universal reference. Calculations were performed using Microsoft Excel (Excel for MAC 2008; Redmond, WA, USA) and the JMP 8.0 software (SAS, Cary, NC, USA).

### Patient data/human glioma samples

A tissue micro array (TMA) developed from formalin‐fixed, paraffin‐embedded (FFPE) tissue samples of 303 patients comprising 16 diffuse astrocytomas (WHO grade II), 35 anaplastic astrocytomas (WHO grade III), and 252 glioblastomas (WHO grade IV) from the tissue bank of the Edinger Institute was analyzed. Furthermore, infiltration zones (*n* = 39), normal‐appearing white matter (NAWM; *n* = 18), and normal‐appearing gray matter (NAGM; *n* = 48) of GBM (*n* = 73) samples were included. In order to minimize quantification bias, randomly selected biopsy sections from patients from the TMA cohort were analyzed (healthy human brain, *n* = 3; diffuse low‐grade glioma, *n* = 14; anaplastic glioma, *n* = 12; and glioblastoma, *n* = 11). Additionally, we analyzed sections (FFPE) of a separate patient cohort consisting of 29 patients with GBM who received bevacizumab during the clinical course (for more detailed patient information, see Appendix Table S1). We compared the primary tumor (treatment‐naive) with tumor tissue after bevacizumab treatment. If available, we additionally compared tumor tissue after radiochemotherapy (*n* = 8) with primary tumors (*n* = 27) and tumor tissue after bevacizumab treatment (*n* = 29). The histopathological diagnoses were performed according to the WHO criteria by board‐certified neuropathologists (MMi, PNH, KHP) (Louis *et al*, 2007). The use of patient material for this study was approved by the local ethics committee (GS‐04/09 and SNO_10‐13).

### Immunohistochemistry of human glioma samples

Glioma and control specimens were fixed in 10% formaldehyde and embedded in paraffin. Brain sections (4 μm) were deparaffinized, and antigen retrieval was performed. For Iba1, sections were pre‐treated in 10 mM citrate buffer (pH 6.0, microwave steamer). After incubation with primary antibodies, appropriate secondary antibodies were applied and cells were visualized using ABC and AEC kits. For CD31, CD3, CD8, CD15, CD20, CAIX, and Ang‐2 IHC, a Ventana Benchmark automated staining system was used. For antigen retrieval, cell conditioning 1 (Citrate buffer solution, Ventana Medical Systems) was used. After incubation with antibodies, signal was detected with Chromo DAB Map kit (Ventana Medical Systems) in case of CD3, with DAB Map kit (Ventana Medical Systems) for CD15, CD20, and α‐SMA and with IHC Ultra Map AP (Ventana Medical Systems) kit for Ang‐2. For double‐immunofluorescence, freshly frozen human glioblastoma, samples were fixed in ice‐cold acetone for 10 min. Antigen retrieval was performed by pre‐incubation in 10 mM citrate buffer (pH 6) for 40 min in a steamer (MultiGourmet, Braun). Slides were incubated with anti‐human Ang‐2 antibody (1:100) and either anti‐human vWF (1:100) or anti‐human Iba1 (1:100) for 1.5 h at RT. After thorough washing steps, detection of primary antibody with respective Alexa Fluor‐labeled secondary antibody was achieved during 1 h at RT. Due to species incompatibility, double labeling with αSMA‐Cy3 (Sigma, 1:200) was achieved by a sequential staining procedure. DAPI was used for nuclear counterstain. All fluorescent images were acquired using a Nikon Eclipse TE‐2000E confocal microscope. The cohort of bevacizumab‐treated patient biopsies was investigated by immunohistochemistry using standard protocols on an automated immunohistochemistry system (Ventana Medical Systems, Tucson, AZ, USA) and the following antibodies: CD68 (1:500; DAKO), CD31 (1:200; DAKO), CSF1R (1:100; Abcam), CD206 (1:100 Abcam), and Ang‐2 (1:200; Pierce Biotechnolgy).

### Analysis of human glioma immunohistochemistry samples

Immunohistochemical stainings were assessed applying a semiquantitative scoring system (Harter *et al*, 2010). Evaluation of the immunohistochemistry preparations and image acquisition was performed using an Olympus BX50 light microscope. The immunohistochemical staining intensity (low = 1, moderate = 2, strong = 3) was multiplied by the proportion of positive vascular cells separately (1–10% = 1, 10–25% = 2, 25–50% = 3, > 50% = 4). The semiquantitative scores were assigned as an ordinal scale response variable and statistical differences among WHO grades or areas were assessed using the nonparametric Wilcoxon test. Patient survival was determined using the median split of expression scores in a Kaplan–Meier analysis. In order to compare the survival curves, we used Wilcoxon and log‐rank test for censored data. In addition, biopsy sections from a subcohort of patients (see Patient data/human glioma samples) were analyzed for CD31^+^ vessels/mm^2^ and Ang‐2^+^ vessels/mm^2^ on serial sections. A significance level of alpha = 0.05 was chosen for all tests. Statistical analysis was performed using JMP 11.0 software (SAS, Cary, NC, USA). The cohort of bevacizumab‐treated patient biopsies (treatment‐naive GBM, post‐temozolomide radiochemotherapy, post‐bevacizumab) was analyzed for the following parameters: vessel density (CD31^+^ vessels per mm^2^), Ang‐2 expression (Ang‐2^+^ vessels per mm^2^), infiltration of CD68^+^, CD206^+^, and CSF1R^+^ cells (per mm^2^). Additionally, we analyzed the number of CD206‐positive cells which surround blood vessels for all three conditions. To assess whether there are differences in oxygenation of the tissue, we analyzed the expression of carboanhydrase IX (CAIX) as a marker for hypoxia and measured the tumor area which was positive for CAIX in relation to the whole tumor area on the tissue sections. Lymphocytes were analyzed as CD3‐ and CD8‐positive cells per mm^2^ and depicted as CD8/CD3 ratio. Analysis was performed using a light microscope with the Stereo Investigator as well as the Fractionator (MicroBrightField Inc, Williston, USA) and analySIS software (Olympus Soft Imaging Solutions, Muenster, Germany). Analyses were performed using GraphPad Prism software (GraphPad Software Inc, La Jolla, CA, USA) and JMP 11.0 software (SAS, Cary, NC, USA).

### ELISA

Angiopoietin serum levels were determined using human Ang‐1 and Ang‐2 BD Quantikine ELISA kits (R&D Systems) according to the manufacturer instructions and as previously described (Reiss *et al*, 2007; Scholz *et al*, 2011).

### Western blot of mouse GBM samples

Mouse brain tumor tissue was snap‐frozen in liquid nitrogen and stored at −80°C prior to use. Frozen tumor tissue was thawed on ice, immediately immersed in 500 μl RIPA buffer with proteinase and phosphatase inhibitors (Roche), and transferred to a Precellys Ceramic Kit tube (Peqlab). The tissue was homogenized twice by a Minilys homogenizer (Peqlab) for 30 s at full speed, followed by centrifugation at 16,000 *g* for 10 min at 4°C. After determining the protein concentration, the protein lysate was denatured in 4× sample buffer (Merck Millipore) and loaded to precast Bis‐Tris gels (Life technologies). Gels were transferred to nitrocellulose membranes and blocked for 1 h with 5% BSA in PBS‐T (PBS/0.1% Tween‐20). The membranes were subsequently incubated with primary antibodies (anti‐mouse Ang‐2, Abcam; anti‐mouse CD31 and anti‐β‐actin, both Santa Cruz) diluted 1:1,000 in the blocking buffer ON at 4°C. Following three washing steps of the nitrocellulose membranes (3 × 10 min with PBS‐T), they were incubated with corresponding secondary antibodies diluted in the blocking buffer for 1 h at RT. Secondary antibodies were coupled to horseradish peroxidase (HRP) for enhanced chemoluminescence (ECL) detection or to a fluorophore. The membranes were subjected to ECL detection if necessary, after washing them 3 × 10 min with PBS‐T. The secondary antibody signals were visualized by Odyssey^™^ imaging device (LI‐COR), which detects both chemiluminescent and fluorescent signals. Quantitation was performed on exported raw tiff files using Image Studio 3.1 software (LI‐COR).

### Flow cytometry of Ang‐2 transgenic brains and GL261 tumors

Normal brains of Ang‐2 DT mice and wild‐type littermates were dissected and immediately transferred in ice‐cold HBSS. Tumors of GL261‐bearing mice were treated the same way. Tumor‐bearing mice were terminated as soon as the first animal of a treatment group (i.e. untreated, AMG386, Aflibercept, or the combination of both) showed symptoms. After gentle mincing (individual brains or tumors were treated separately), the tissue was incubated in a HBSS solution containing Collagenase P (0.2 mg/ml), Dispase II (0.8 mg/ml), DNase I (0.01 mg/ml), and Collagenase A (0.3 mg/ml) for 60 min at 37°C under gentle rocking (as previously described by Lee *et al*, 2014). Digestion was stopped by adding FBS on ice. The supernatants were centrifuged at 250 *g* for 10 min at 4°C. The pellet was resuspended in 25% BSA/PBS for myelin removal. Following a centrifugation step at 3,000 *g* for 30 min at 4°C, the myelin containing supernatant was discarded. The sample was then resuspended in 1 ml HBSS and filtered through a 40‐μm mesh, followed by a washing step in HBSS (centrifugation at 250 *g* for 10 min at 4°C). The pellet was resuspended in 1 ml red blood cell lysis buffer (Roche) and incubated for 10 min at RT for lysis of erythrocytes. Subsequently, 2 ml FACS buffer (5% FCS in PBS) was added and cells were centrifuged at 250 *g* for 10 min at 4°C. The cells were pre‐incubated with rat anti‐mouse FcγIII/II receptor (CD16/CD32)‐blocking antibodies (≤ 1 μg/million cells/100 μl; BD) for 5 min at 4°C and then stained with the fluorochrome‐conjugated antibodies (0.25–1 μg; listed below). Following two washes, DAPI (4′,6‐diamidino‐2‐phenylindole; Invitrogen) was added for live gating and cells were acquired on a FACSCanto^™^ II flow cytometer (BD) using Diva software (BD) and further analyzed using FlowJo analytical software (FlowJo version 10.0.8, LLC). Background fluorescence levels were determined by Fluorescence Minus One (FMO).

Antibodies applied for flow cytometry were as follows: CD45 PercP Cy5.5 (clone 30‐F11, BD Biosciences), Gr‐1 APC‐Cy7 (clone RB6‐8C5, BD Biosciences), CD11b APC (clone M1/70, BD), F4/80 FITC (clone BM8, eBioscence), CD206 PE‐Cy7 (clone C068C2, Biolegend), and MHC class II PE (clone M5/114.15.2, BD Biosciences).

### Statistical analyses

Quantitative and statistical analyses were performed using GraphPad Prism software (GraphPad Software, Inc) and JMP 11.0 software (SAS, Cary, NC, USA). Statistical tests applied are indicated in the individual figure legends. For all non‐survival statistical analyses of two experimental groups an unpaired, two‐tailed Student's *t*‐test was performed. For multiple comparison, analyses of more than two groups ANOVA (one‐ or two‐way) followed by Tukey post‐test, or Kruskal–Wallis test followed by Dunn's post‐test were used. Kaplan–Meier survival curves were analyzed using the log‐rank and nonparametric Wilcoxon test. *P*‐values < 0.05 were considered statistically significant and indicated with asterisks (*P < 0.05; **P < 0.01; ***P < 0.005). Data are represented as mean ± SEM if not indicated otherwise. Please see Appendix Table S2 for individual *P*‐values.

## Author contributions

YR and KHP were involved in study conception and design. YR, KHP, AS, PNH, and MMi developed methodology. AS, SC, PNH, BHY, SG, MY, MDT, KS, PB, and MMi were involved in data acquisition. YR, KHP, AS, SC, PNH, and MMi analyzed and interpreted data. YR and KHP wrote, reviewed, and/or revised the manuscript. MG, UH, DK, MMe, AW, MT, RG, MD, CB, JS, JT, OB, JPS, JTF, EU, and MMi provided administrative, technical, or material support. YR and KHP supervised the study.

## Conflict of interest

Research support from Amgen (KHP), Amgen Europe Advisory Board Member (KHP).

**The paper explained**

**Problem**

Current evidence suggests that failure of anti‐angiogenic or radiotherapy in patients is associated with an influx of “rebound” myeloid cells that preferentially switch to a tumor promoting M2 polarization profile in the expense of a tumoricidal M1 phenotype. However, the exact molecular mechanisms causing recruitment of myeloid cells into tumors in the course of therapy, the factors that control M1 versus M2 polarization, and the precise role of tumor‐associated macrophages for anti‐angiogenic therapy resistance are only partially understood. Unraveling these mechanisms and the assessment of combinatorial therapies, in particular the combination of novel anti‐angiogenic therapies with inhibitors of myeloid cell infiltration/polarization is therefore of considerable interest in order to pave the way for novel therapeutic options for the treatment of glioblastoma.

**Results**

In a transgenic mouse model for inducible, endothelial cell‐specific expression of Ang‐2, we observed that Ang‐2 increased myeloid cell infiltration in glioma models, whereas in glioma‐bearing syngeneic wild‐type mice angiogenesis inhibitors blocked myeloid cell infiltration but—depending of the type of inhibitors used—induced a “switch” in macrophage polarization in favour of the pro‐angiogenic, anti‐inflammatory M2‐phenotype that supports tumor progression. We provide evidence that the combined inhibition of angiopoietin and VEGF signaling may obliterate resistance to VEGF monotherapy caused by upregulation of Ang‐2 in endothelial cells, accompanied by the presence of alternatively polarized perivascular macrophages.

**Impact**

Our findings imply a novel role for endothelial cells in therapy resistance and identify endothelial cell/myeloid cell crosstalk mediated by Ang‐2 as a potential resistance mechanism. Therefore, combining VEGF blockade with inhibition of Ang‐2 may potentially overcome resistance to bevacizumab therapy. In addition, CD206^+^ (M2‐like) macrophages were identified as potential novel targets in bevacizumab‐resistant GBM.

## Supporting information



Appendix

Expanded View Figures PDF

Review Process File

Source Data for Figure 1C

## References

[emmm201505505-bib-0001] Augustin HG , Koh GY , Thurston G , Alitalo K (2009) Control of vascular morphogenesis and homeostasis through the angiopoietin‐Tie system. Nat Rev Mol Cell Biol 10: 165–177 19234476 10.1038/nrm2639

[emmm201505505-bib-0002] Avraham HK , Jiang S , Fu Y , Nakshatri H , Ovadia H , Avraham S (2014) Angiopoietin‐2 mediates blood‐brain barrier impairment and colonization of triple‐negative breast cancer cells in brain. J Pathol 232: 369–381 24421076 10.1002/path.4304

[emmm201505505-bib-0003] Avraham‐Davidi I , Yona S , Grunewald M , Landsman L , Cochain C , Silvestre JS , Mizrahi H , Faroja M , Strauss‐Ayali D , Mack M *et al* (2013) On‐site education of VEGF‐recruited monocytes improves their performance as angiogenic and arteriogenic accessory cells. J Exp Med 210: 2611–2625 24166715 10.1084/jem.20120690 PMC3832929

[emmm201505505-bib-0004] Bais C , Wu X , Yao J , Yang S , Crawford Y , McCutcheon K , Tan C , Kolumam G , Vernes J‐M , Eastham‐Anderson J *et al* (2010) PlGF blockade does not inhibit angiogenesis during primary tumor growth. Cell 141: 166–177 20371352 10.1016/j.cell.2010.01.033

[emmm201505505-bib-0005] Batchelor TT , Reardon DA , de Groot JF , Wick W , Weller M (2014) Antiangiogenic therapy for glioblastoma: current status and future prospects. Clin Cancer Res 20: 5612–5619 25398844 10.1158/1078-0432.CCR-14-0834 PMC4234180

[emmm201505505-bib-0006] Biswas SK , Allavena P , Mantovani A (2013) Tumor‐associated macrophages: functional diversity, clinical significance, and open questions. Semin Immunopathol 35: 585–600 23657835 10.1007/s00281-013-0367-7

[emmm201505505-bib-0007] Brauer MJ , Zhuang G , Schmidt M , Yao J , Wu X , Kaminker JS , Jurinka SS , Kolumam G , Chung AS , Jubb A *et al* (2013) Identification and analysis of *in vivo* VEGF downstream markers link VEGF pathway activity with efficacy of anti‐VEGF therapies. Clin Cancer Res 19: 3681–3692 23685835 10.1158/1078-0432.CCR-12-3635

[emmm201505505-bib-0008] Brown JL , Cao ZA , Pinzon‐Ortiz M , Kendrew J , Reimer C , Wen S , Zhou JQ , Tabrizi M , Emery S , McDermott B *et al* (2010) A human monoclonal anti‐ANG2 antibody leads to broad antitumor activity in combination with VEGF inhibitors and chemotherapy agents in preclinical models. Mol Cancer Ther 9: 145–156 20053776 10.1158/1535-7163.MCT-09-0554

[emmm201505505-bib-0009] Burrell K , Singh S , Jalali S , Hill RP , Zadeh G (2014) VEGF regulates region‐specific localization of perivascular bone marrow‐derived cells in glioblastoma. Cancer Res 74: 3727–3739 24820020 10.1158/0008-5472.CAN-13-3119

[emmm201505505-bib-0010] Carmeliet P , Jain RK (2011) Molecular mechanisms and clinical applications of angiogenesis. Nature 473: 298–307 21593862 10.1038/nature10144 PMC4049445

[emmm201505505-bib-0011] Chinot OL , Wick W , Mason W , Henriksson R , Saran F , Nishikawa R , Carpentier AF , Hoang‐Xuan K , Kavan P , Cernea D *et al* (2014) Bevacizumab plus radiotherapy‐temozolomide for newly diagnosed glioblastoma. N Engl J Med 370: 709–722 24552318 10.1056/NEJMoa1308345

[emmm201505505-bib-0012] Chung AS , Wu X , Zhuang G , Ngu H , Kasman I , Zhang J , Vernes J‐M , Jiang Z , Meng YG , Peale FV *et al* (2013) An interleukin‐17‐mediated paracrine network promotes tumor resistance to anti‐angiogenic therapy. Nat Med 19: 1114–1123 23913124 10.1038/nm.3291

[emmm201505505-bib-0013] Coffelt SB , Tal AO , Scholz A , De Palma M , Patel S , Urbich C , Biswas SK , Murdoch C , Plate KH , Reiss Y *et al* (2010) Angiopoietin‐2 regulates gene expression in TIE2‐expressing monocytes and augments their inherent proangiogenic functions. Cancer Res 70: 5270–5280 20530679 10.1158/0008-5472.CAN-10-0012

[emmm201505505-bib-0014] Coffelt SB , Chen Y‐Y , Muthana M , Welford AF , Tal AO , Scholz A , Plate KH , Reiss Y , Murdoch C , De Palma M *et al* (2011) Angiopoietin 2 stimulates TIE2‐expressing monocytes to suppress T cell activation and to promote regulatory T cell expansion. J Immunol 186: 4183–4190 21368233 10.4049/jimmunol.1002802

[emmm201505505-bib-0015] Cohen MH , Shen YL , Keegan P , Pazdur R (2009) FDA drug approval summary: bevacizumab (Avastin) as treatment of recurrent glioblastoma multiforme. Oncologist 14: 1131–1138 19897538 10.1634/theoncologist.2009-0121

[emmm201505505-bib-0016] Coxon A , Bready J , Min H , Kaufman S , Leal J , Yu D , Lee TA , Sun JR , Estrada J , Bolon B *et al* (2010) Context‐dependent role of angiopoietin‐1 inhibition in the suppression of angiogenesis and tumor growth: implications for AMG 386, an angiopoietin‐1/2‐neutralizing peptibody. Mol Cancer Ther 9: 2641–2651 20937592 10.1158/1535-7163.MCT-10-0213 PMC4430860

[emmm201505505-bib-0017] Daly C , Eichten A , Castanaro C , Pasnikowski E , Adler A , Lalani AS , Papadopoulos N , Kyle AH , Minchinton AI , Yancopoulos GD *et al* (2013) Angiopoietin‐2 functions as a Tie2 agonist in tumor models, where it limits the effects of VEGF inhibition. Cancer Res 73: 108–118 23149917 10.1158/0008-5472.CAN-12-2064

[emmm201505505-bib-0018] De Palma M , Lewis CE (2013) Macrophage regulation of tumor responses to anticancer therapies. Cancer Cell 23: 277–286 23518347 10.1016/j.ccr.2013.02.013

[emmm201505505-bib-0019] Eklund L , Saharinen P (2013) Angiopoietin signaling in the vasculature. Exp Cell Res 319: 1271–1280 23500414 10.1016/j.yexcr.2013.03.011

[emmm201505505-bib-0020] Falcón BL , Hashizume H , Koumoutsakos P , Chou J , Bready JV , Coxon A , Oliner JD , McDonald DM (2009) Contrasting actions of selective inhibitors of angiopoietin‐1 and angiopoietin‐2 on the normalization of tumor blood vessels. Am J Pathol 175: 2159–2170 19815705 10.2353/ajpath.2009.090391 PMC2774078

[emmm201505505-bib-0021] Fiedler U , Reiss Y , Scharpfenecker M , Grunow V , Koidl S , Thurston G , Gale NW , Witzenrath M , Rosseau S , Suttorp N *et al* (2006) Angiopoietin‐2 sensitizes endothelial cells to TNF‐alpha and has a crucial role in the induction of inflammation. Nat Med 12: 235–239 16462802 10.1038/nm1351

[emmm201505505-bib-0022] Fischer C , Jonckx B , Mazzone M , Zacchigna S , Loges S , Pattarini L , Chorianopoulos E , Liesenborghs L , Koch M , De Mol M *et al* (2007) Anti‐PlGF inhibits growth of VEGF(R)‐inhibitor‐resistant tumors without affecting healthy vessels. Cell 131: 463–475 17981115 10.1016/j.cell.2007.08.038

[emmm201505505-bib-0023] Gerald D , Chintharlapalli S , Augustin HG , Benjamin LE (2013) Angiopoietin‐2: an attractive target for improved antiangiogenic tumor therapy. Cancer Res 73: 1649–1657 23467610 10.1158/0008-5472.CAN-12-4697

[emmm201505505-bib-0024] Gilbert MR , Dignam JJ , Armstrong TS , Wefel JS , Blumenthal DT , Vogelbaum MA , Colman H , Chakravarti A , Pugh S , Won M *et al* (2014) A randomized trial of bevacizumab for newly diagnosed glioblastoma. N Engl J Med 370: 699–708 24552317 10.1056/NEJMoa1308573 PMC4201043

[emmm201505505-bib-0025] Grunewald M , Avraham I , Dor Y , Bachar‐Lustig E , Itin A , Jung S , Yung S , Chimenti S , Landsman L , Abramovitch R *et al* (2006) VEGF‐induced adult neovascularization: recruitment, retention, and role of accessory cells. Cell 124: 175–189 16413490 10.1016/j.cell.2005.10.036

[emmm201505505-bib-0026] Harter PN , Bunz B , Dietz K , Hoffmann K , Meyermann R , Mittelbronn M (2010) Spatio‐temporal deleted in colorectal cancer (DCC) and netrin‐1 expression in human foetal brain development. Neuropathol Appl Neurobiol 36: 623–635 20609112 10.1111/j.1365-2990.2010.01100.x

[emmm201505505-bib-0027] Hashizume H , Falcon BL , Kuroda T , Baluk P , Coxon A , Yu D , Bready JV , Oliner JD , McDonald DM (2010) Complementary actions of inhibitors of angiopoietin‐2 and VEGF on tumor angiogenesis and growth. Cancer Res 70: 2213–2223 20197469 10.1158/0008-5472.CAN-09-1977 PMC2840050

[emmm201505505-bib-0028] Helfrich I , Edler L , Sucker A , Thomas M , Christian S , Schadendorf D , Augustin HG (2009) Angiopoietin‐2 levels are associated with disease progression in metastatic malignant melanoma. Clin Cancer Res 15: 1384–1392 19228739 10.1158/1078-0432.CCR-08-1615

[emmm201505505-bib-0029] Holash J , Maisonpierre PC , Compton D , Boland P , Alexander CR , Zagzag D , Yancopoulos GD , Wiegand SJ (1999) Vessel cooption, regression, and growth in tumors mediated by angiopoietins and VEGF. Science 284: 1994–1998 10373119 10.1126/science.284.5422.1994

[emmm201505505-bib-0030] Holash J , Davis S , Papadopoulos N , Croll SD , Ho L , Russell M , Boland P , Leidich R , Hylton D , Burova E *et al* (2002) VEGF‐Trap: a VEGF blocker with potent antitumor effects. Proc Natl Acad Sci USA 99: 11393–11398 12177445 10.1073/pnas.172398299 PMC123267

[emmm201505505-bib-0031] Holopainen T , Saharinen P , D'Amico G , Lampinen A , Eklund L , Sormunen R , Anisimov A , Zarkada G , Lohela M , Heloterä H *et al* (2012) Effects of angiopoietin‐2‐blocking antibody on endothelial cell‐cell junctions and lung metastasis. J Natl Cancer Inst 104: 461–475 22343031 10.1093/jnci/djs009 PMC3309130

[emmm201505505-bib-0032] Huang H , Lai J‐Y , Do J , Liu D , Li L , Del Rosario J , Doppalapudi VR , Pirie‐Shepherd S , Levin N , Bradshaw C *et al* (2011) Specifically targeting angiopoietin‐2 inhibits angiogenesis, Tie2‐expressing monocyte infiltration, and tumor growth. Clin Cancer Res 17: 1001–1011 21233403 10.1158/1078-0432.CCR-10-2317

[emmm201505505-bib-0033] Huang Y , Yuan J , Righi E , Kamoun WS , Ancukiewicz M , Nezivar J , Santosuosso M , Martin JD , Martin MR , Vianello F *et al* (2012) Vascular normalizing doses of antiangiogenic treatment reprogram the immunosuppressive tumor microenvironment and enhance immunotherapy. Proc Natl Acad Sci USA 109: 17561–17566 23045683 10.1073/pnas.1215397109 PMC3491458

[emmm201505505-bib-0034] Hughes R , Qian B‐Z , Rowan C , Muthana M , Keklikoglou I , Olson OC , Tazzyman S , Danson S , Addison C , Clemons M *et al* (2015) Perivascular M2 macrophages stimulate tumor relapse after chemotherapy. Cancer Res 75: 3479–3491 26269531 10.1158/0008-5472.CAN-14-3587 PMC5024531

[emmm201505505-bib-0035] Hurwitz H , Fehrenbacher L , Novotny W , Cartwright T , Hainsworth J , Heim W , Berlin J , Baron A , Griffing S , Holmgren E *et al* (2004) Bevacizumab plus irinotecan, fluorouracil, and leucovorin for metastatic colorectal cancer. N Engl J Med 350: 2335–2342 15175435 10.1056/NEJMoa032691

[emmm201505505-bib-0036] Jain RK (2005) Normalization of tumor vasculature: an emerging concept in antiangiogenic therapy. Science 307: 58–62 15637262 10.1126/science.1104819

[emmm201505505-bib-0037] Kerber M , Reiss Y , Wickersheim A , Jugold M , Kiessling F , Heil M , Tchaikovski V , Waltenberger J , Shibuya M , Plate KH *et al* (2008) Flt‐1 signaling in macrophages promotes glioma growth *in vivo* . Cancer Res 68: 7342–7351 18794121 10.1158/0008-5472.CAN-07-6241

[emmm201505505-bib-0038] Kienast Y , Klein C , Scheuer W , Raemsch R , Lorenzon E , Bernicke D , Herting F , Yu S , The HH , Martarello L *et al* (2013) Ang‐2‐VEGF‐A CrossMab, a novel bispecific human IgG1 antibody blocking VEGF‐A and Ang‐2 functions simultaneously, mediates potent antitumor, antiangiogenic, and antimetastatic efficacy. Clin Cancer Res 19: 6730–6740 24097868 10.1158/1078-0432.CCR-13-0081

[emmm201505505-bib-0039] Koh YJ , Kim HZ , Hwang S‐I , Lee JE , Oh N , Jung K , Kim M , Kim KE , Kim H , Lim N‐K *et al* (2010) Double antiangiogenic protein, DAAP, targeting VEGF‐A and angiopoietins in tumor angiogenesis, metastasis, and vascular leakage. Cancer Cell 18: 171–184 20708158 10.1016/j.ccr.2010.07.001

[emmm201505505-bib-0040] Lee M , Kiefel H , LaJevic MD , Macauley MS , Kawashima H , O'Hara E , Pan J , Paulson JC , Butcher EC (2014) Transcriptional programs of lymphoid tissue capillary and high endothelium reveal control mechanisms for lymphocyte homing. Nat Immunol 15: 982–995 25173345 10.1038/ni.2983 PMC4222088

[emmm201505505-bib-0041] Leow CC , Coffman K , Inigo I , Breen S , Czapiga M , Soukharev S , Gingles N , Peterson N , Fazenbaker C , Woods R *et al* (2012) MEDI3617, a human anti‐angiopoietin 2 monoclonal antibody, inhibits angiogenesis and tumor growth in human tumor xenograft models. Int J Oncol 40: 1321–1330 22327175 10.3892/ijo.2012.1366

[emmm201505505-bib-0042] Liontos M , Lykka M , Dimopoulos M‐A , Bamias A (2014) Profile of trebananib (AMG386) and its potential in the treatment of ovarian cancer. Onco Targets Ther 7: 1837–1845 25336975 10.2147/OTT.S65522 PMC4199819

[emmm201505505-bib-0043] Louis DN , Ohgaki H , Wiestler OD , Cavenee WK , Burger PC , Jouvet A , Scheithauer BW , Kleihues P (2007) The 2007 WHO classification of tumours of the central nervous system. Acta Neuropathol 114: 97–109 17618441 10.1007/s00401-007-0243-4 PMC1929165

[emmm201505505-bib-0044] Mazzieri R , Pucci F , Moi D , Zonari E , Ranghetti A , Berti A , Politi LS , Gentner B , Brown JL , Naldini L *et al* (2011) Targeting the ANG2/TIE2 axis inhibits tumor growth and metastasis by impairing angiogenesis and disabling rebounds of proangiogenic myeloid cells. Cancer Cell 19: 512–526 21481792 10.1016/j.ccr.2011.02.005

[emmm201505505-bib-0045] Monk BJ , Poveda A , Vergote I , Raspagliesi F , Fujiwara K , Bae D‐S , Oaknin A , Ray‐Coquard I , Provencher DM , Karlan BY *et al* (2014) Anti‐angiopoietin therapy with trebananib for recurrent ovarian cancer (TRINOVA‐1): a randomised, multicentre, double‐blind, placebo‐controlled phase 3 trial. Lancet Oncol 15: 799–808 24950985 10.1016/S1470-2045(14)70244-X

[emmm201505505-bib-0046] Motz GT , Santoro SP , Wang L‐P , Garrabrant T , Lastra RR , Hagemann IS , Lal P , Feldman MD , Benencia F , Coukos G (2014) Tumor endothelium FasL establishes a selective immune barrier promoting tolerance in tumors. Nat Med 20: 607–615 24793239 10.1038/nm.3541 PMC4060245

[emmm201505505-bib-0047] Ohgaki H , Kleihues P (2005) Epidemiology and etiology of gliomas. Acta Neuropathol 109: 93–108 15685439 10.1007/s00401-005-0991-y

[emmm201505505-bib-0048] Oliner J , Min H , Leal J , Yu D , Rao S , You E , Tang X , Kim H , Meyer S , Han SJ *et al* (2004) Suppression of angiogenesis and tumor growth by selective inhibition of angiopoietin‐2. Cancer Cell 6: 507–516 15542434 10.1016/j.ccr.2004.09.030

[emmm201505505-bib-0049] Park JH , Park KJ , Kim YS , Sheen SS , Lee KS , Lee HN , Oh YJ , Hwang SC (2007) Serum angiopoietin‐2 as a clinical marker for lung cancer. Chest 132: 200–206 17505039 10.1378/chest.06-2915

[emmm201505505-bib-0050] Phillips HS , Kharbanda S , Chen R , Forrest WF , Soriano RH , Wu TD , Misra A , Nigro JM , Colman H , Soroceanu L *et al* (2006) Molecular subclasses of high‐grade glioma predict prognosis, delineate a pattern of disease progression, and resemble stages in neurogenesis. Cancer Cell 9: 157–173 16530701 10.1016/j.ccr.2006.02.019

[emmm201505505-bib-0051] Pyonteck SM , Akkari L , Schuhmacher AJ , Bowman RL , Sevenich L , Quail DF , Olson OC , Quick ML , Huse JT , Teijeiro V *et al* (2013) CSF‐1R inhibition alters macrophage polarization and blocks glioma progression. Nat Med 19: 1264–1272 24056773 10.1038/nm.3337 PMC3840724

[emmm201505505-bib-0052] Quail DF , Joyce JA (2013) Microenvironmental regulation of tumor progression and metastasis. Nat Med 19: 1423–1437 24202395 10.1038/nm.3394 PMC3954707

[emmm201505505-bib-0053] Reiss Y , Droste J , Heil M , Tribulova S , Schmidt MHH , Schaper W , Dumont DJ , Plate KH (2007) Angiopoietin‐2 impairs revascularization after limb ischemia. Circ Res 101: 88–96 17540977 10.1161/CIRCRESAHA.106.143594

[emmm201505505-bib-0054] Reiss Y , Scholz A , Plate KH (2015) The angiopoietin—tie system: common signaling pathways for angiogenesis, cancer, and inflammation. Springer: Endothel Signal Dev Dis 13: 313–328

[emmm201505505-bib-0055] Ries CH , Cannarile MA , Hoves S , Benz J , Wartha K , Runza V , Rey‐Giraud F , Pradel LP , Feuerhake F , Klaman I *et al* (2014) Targeting tumor‐associated macrophages with anti‐CSF‐1R antibody reveals a strategy for cancer therapy. Cancer Cell 25: 846–859 24898549 10.1016/j.ccr.2014.05.016

[emmm201505505-bib-0056] Rigamonti N , Kadioglu E , Keklikoglou I , Wyser Rmili C , Leow CC , De Palma M (2014) Role of angiopoietin‐2 in adaptive tumor resistance to VEGF signaling blockade. Cell Rep 8: 696–706 25088418 10.1016/j.celrep.2014.06.059

[emmm201505505-bib-0057] Sallinen H , Heikura T , Koponen J , Kosma V‐M , Heinonen S , Ylä‐Herttuala S , Anttila M (2014) Serum angiopoietin‐2 and soluble VEGFR‐2 levels predict malignancy of ovarian neoplasm and poor prognosis in epithelial ovarian cancer. BMC Cancer 14: 696 25245329 10.1186/1471-2407-14-696 PMC4179851

[emmm201505505-bib-0058] Sandmann T , Bourgon R , Garcia J , Li C , Cloughesy T , Chinot OL , Wick W , Nishikawa R , Mason W , Henriksson R *et al* (2015) Patients with proneural glioblastoma may derive overall survival benefit from the addition of bevacizumab to first‐line radiotherapy and temozolomide: retrospective analysis of the AVAglio trial. J Clin Oncol 33: 2735–2744 26124478 10.1200/JCO.2015.61.5005 PMC5015426

[emmm201505505-bib-0059] Schneider K , Weyerbrock A , Doostkam S , Plate K , Machein MR (2015) Lack of evidence for PlGF mediating the tumor resistance after anti‐angiogenic therapy in malignant gliomas. J Neurooncol 121: 269–278 25370707 10.1007/s11060-014-1647-3

[emmm201505505-bib-0060] Scholz A , Lang V , Henschler R , Czabanka M , Vajkoczy P , Chavakis E , Drynski J , Harter PN , Mittelbronn M , Dumont DJ *et al* (2011) Angiopoietin‐2 promotes myeloid cell infiltration in a β₂‐integrin‐dependent manner. Blood 118: 5050–5059 21868579 10.1182/blood-2011-03-343293

[emmm201505505-bib-0061] Scholz A , Plate KH , Reiss Y (2015) Angiopoietin‐2: a multifaceted cytokine that functions in both angiogenesis and inflammation. Ann N Y Acad Sci 1347: 45–51 25773744 10.1111/nyas.12726

[emmm201505505-bib-0062] Shojaei F , Wu X , Malik AK , Zhong C , Baldwin ME , Schanz S , Fuh G , Gerber H‐P , Ferrara N (2007) Tumor refractoriness to anti‐VEGF treatment is mediated by CD11b+Gr1+ myeloid cells. Nat Biotechnol 25: 911–920 17664940 10.1038/nbt1323

[emmm201505505-bib-0063] Stratmann A , Risau W , Plate KH (1998) Cell type‐specific expression of angiopoietin‐1 and angiopoietin‐2 suggests a role in glioblastoma angiogenesis. Am J Pathol 153: 1459–1466 9811337 10.1016/S0002-9440(10)65733-1 PMC1853417

[emmm201505505-bib-0064] Stupp R , Mason WP , van den Bent MJ , Weller M , Fisher B , Taphoorn MJB , Belanger K , Brandes AA , Marosi C , Bogdahn U *et al* (2005) Radiotherapy plus concomitant and adjuvant temozolomide for glioblastoma. N Engl J Med 352: 987–996 15758009 10.1056/NEJMoa043330

[emmm201505505-bib-0065] Taal W , Oosterkamp HM , Walenkamp AME , Dubbink HJ , Beerepoot LV , Hanse MCJ , Buter J , Honkoop AH , Boerman D , de Vos FYF *et al* (2014) Single‐agent bevacizumab or lomustine versus a combination of bevacizumab plus lomustine in patients with recurrent glioblastoma (BELOB trial): a randomised controlled phase 2 trial. Lancet Oncol 15: 943–953 25035291 10.1016/S1470-2045(14)70314-6

[emmm201505505-bib-0066] Tabruyn SP , Colton K , Morisada T , Fuxe J , Wiegand SJ , Thurston G , Coyle AJ , Connor J , McDonald DM (2010) Angiopoietin‐2‐driven vascular remodeling in airway inflammation. Am J Pathol 177: 3233–3244 20952594 10.2353/ajpath.2010.100059 PMC2993300

[emmm201505505-bib-0067] Thomas M , Kienast Y , Scheuer W , Bähner M , Kaluza K , Gassner C , Herting F , Brinkmann U , Seeber S , Kavlie A *et al* (2013) A novel angiopoietin‐2 selective fully human antibody with potent anti‐tumoral and anti‐angiogenic efficacy and superior side effect profile compared to Pan‐Angiopoietin‐1/‐2 inhibitors. PLoS ONE 8: e54923 23405099 10.1371/journal.pone.0054923 PMC3566157

[emmm201505505-bib-0068] Venneri MA , De Palma M , Ponzoni M , Pucci F , Scielzo C , Zonari E , Mazzieri R , Doglioni C , Naldini L (2007) Identification of proangiogenic TIE2‐expressing monocytes (TEMs) in human peripheral blood and cancer. Blood 109: 5276–5285 17327411 10.1182/blood-2006-10-053504

[emmm201505505-bib-0069] Verhaak RGW , Hoadley KA , Purdom E , Wang V , Qi Y , Wilkerson MD , Miller CR , Ding L , Golub T , Mesirov JP *et al* (2010) Integrated genomic analysis identifies clinically relevant subtypes of glioblastoma characterized by abnormalities in PDGFRA, IDH1, EGFR, and NF1. Cancer Cell 17: 98–110 20129251 10.1016/j.ccr.2009.12.020 PMC2818769

[emmm201505505-bib-0070] Welti J , Loges S , Dimmeler S , Carmeliet P (2013) Recent molecular discoveries in angiogenesis and antiangiogenic therapies in cancer. J Clin Invest 123: 3190–3200 23908119 10.1172/JCI70212 PMC3726176

[emmm201505505-bib-0071] Winkler F , Kozin SV , Tong RT , Chae S‐S , Booth MF , Garkavtsev I , Xu L , Hicklin DJ , Fukumura D , di Tomaso E *et al* (2004) Kinetics of vascular normalization by VEGFR2 blockade governs brain tumor response to radiation: role of oxygenation, angiopoietin‐1, and matrix metalloproteinases. Cancer Cell 6: 553–563 15607960 10.1016/j.ccr.2004.10.011

[emmm201505505-bib-0072] Zagzag D , Hooper A , Friedlander DR , Chan W , Holash J , Wiegand SJ , Yancopoulos GD , Grumet M (1999) *In situ* expression of angiopoietins in astrocytomas identifies angiopoietin‐2 as an early marker of tumor angiogenesis. Exp Neurol 159: 391–400 10506510 10.1006/exnr.1999.7162

