# Supplementary material for: Endothelial cell‐derived angiopoietin‐2 is a therapeutic target in treatment‐naive and bevacizumab‐resistant glioblastoma
Source: EMBO Mol Med. 2015 Dec 14;8(1):39–57. doi: 10.15252/emmm.201505505 (PMC4718155; doi:10.15252/emmm.201505505)
Supplement: Supplementary file 1 — Appendix [file EMMM-8-39-s001.pdf]

Endothelial cell-derived Angiopoietin-2 is a therapeutic target in treatment-naive and Bevacizumab-resistant glioblastoma, A. Scholz et al., Appendix

Table of contents:

Appendix Table S1

Appendix Table S2

Appendix Table S1: Clinical data of the GBM matched biopsy cohort

Patient ID	Age at diagnosis (years)	Sex	MGMT status	Bev treatment (days)	Last Bev therapy prior to histology (days)
1	51	m	U	91	179
2	30	m	U	407	204
3	52	m	U	84	35
4	41	f	U	166	113
5	56	m	n.a.	288	45
6	47	m	U	214	133
7	39	m	M	42	21
8	41	m	n.a.	528	23
9	32	m	M	154	53
10	63	m	U	336	64
11	72	f	U	392	37
12	70	f	U	258	34
13	63	f	U	280	33
14	46	f	U	170	96
15	62	f	U	210	14
16	43	m	U	140	31
17	45	f	U	281	34
18	49	m	n.a.	84	32
19	65	m	n.a.	62	36
20	66	f	M	293	56
21	55	m	U	n.a.	150
22	51	m	U	90	30
24	65	m	U	180	267
25	53	m	n.a.	114	27
26	41	m	U	217	23
27	49	m	M	160	16
28	59	m	U	n.a.	104
29	55	f	M	326	40
30	62	m	M	117	23

M = male, f = female, n.a = not available, MGMT = O6-Methylguanin-DNA-Methyltransferase, U = unmethylated, M = methylated, Bev = Bevacizumab

Appendix Table S2: P values Figures, Expanded View Figures

	P value	
**Figure 1 D**		
Kruskal-Wallis (Dunn's post test)		
Normal brain vs. Diffuse low grade glioma	> 0,9999	
Normal brain vs. Anaplastic astrocytoma	0,1497	
Normal brain vs. Glioblastoma	0,0006	
Diffuse low grade glioma vs. Anaplastic astrocytoma	0,582	
Diffuse low grade glioma vs. Glioblastoma	< 0,0001	
Anaplastic astrocytoma vs. Glioblastoma	0,0519	
**Figure 2 B (CD31)**		
Unpaired t test		
P value	0,0277	
**Figure 2 B (Desmin)**		
Unpaired t test		
P value	0,0425	
**Figure 2 D**		
Unpaired t test		
P value	0,0091	
**Figure 2 F**		
Unpaired t test		
P value	0,0017	
**Figure 3 B (Iba1)**		
Kruskal-Wallis (Dunn's post test)		
normal brain vs. low grade Glioma	0,8432	
normal brain vs. anaplastic Astrocytoma	0,0958	
normal brain vs. Glioblastoma	0,0008	
low grade Glioma vs. anaplastic Astrocytoma	> 0,9999	

low grade Glioma vs. Glioblastoma	0,0179	
anaplastic Astrocytoma vs. Glioblastoma	> 0,9999	
**Figure 3 B (CD15)**		
Kruskal-Wallis (Dunn's post test)		
normal brain vs. low grade Glioma	0,5997	
normal brain vs. anaplastic Astrocytoma	0,0341	
normal brain vs. Glioblastoma	< 0,0001	
low grade Glioma vs. anaplastic Astrocytoma	0,7896	
low grade Glioma vs. Glioblastoma	0,005	
anaplastic Astrocytoma vs. Glioblastoma	0,7476	
**Figure 3 B (CD3)**		
Kruskal-Wallis (Dunn's post test)		
normal brain vs. low grade Glioma	> 0,9999	
normal brain vs. anaplastic Astrocytoma	0,2333	
normal brain vs. Glioblastoma	0,0109	
low grade Glioma vs. anaplastic Astrocytoma	0,1487	
low grade Glioma vs. Glioblastoma	0,0006	
anaplastic Astrocytoma vs. Glioblastoma	> 0,9999	
**Figure 4 B (CD31)**		
Kruskal-Wallis (Dunn's post test)		
Control vs. AMG386	0,4935	
Control vs. Aflibercept	0,3661	
Control vs. Aflibercept + AMG386	< 0,0001	
AMG386 vs. Aflibercept	> 0,9999	
AMG386 vs. Aflibercept + AMG386	0,0213	
Aflibercept vs. Aflibercept + AMG386	0,0141	
**Figure 4 B (Desmin)**		
Kruskal-Wallis (Dunn's post test)		
Control vs. AMG386	0,0013	
Control vs. Aflibercept	< 0,0001	

Control vs. Aflibercept + AMG386	0,0432	
AMG386 vs. Aflibercept	> 0,9999	
AMG386 vs. Aflibercept + AMG386	> 0,9999	
Aflibercept vs. Aflibercept + AMG386	0,6722	
**Figure 4 D**		
one-way ANOVA (Tukey's post test)		
Control vs. AMG386	0,2356	
Control vs. Aflibercept	0,0068	
Control vs. Aflibercept + AMG386	0,034	
AMG386 vs. Aflibercept	0,116	
AMG386 vs. Aflibercept + AMG386	0,5378	
Aflibercept vs. Aflibercept + AMG386	0,6353	
**Figure 4 E**		
one-way ANOVA (Tukey's post test)		
Control vs. AMG386	0,5994	
Control vs. Aflibercept	0,047	
Control vs. Aflibercept + AMG386	0,0017	
AMG386 vs. Aflibercept	0,4422	
AMG386 vs. Aflibercept + AMG386	0,0245	
Aflibercept vs. Aflibercept + AMG386	0,2332	
**Figure 4 H (F4/80)**		
one-way ANOVA (Tukey's post test)		
control vs. AMG386	< 0,0001	
control vs. Zaltrap	< 0,0001	
control vs. AMG386 + Zaltrap	< 0,0001	
AMG386 vs. Zaltrap	0,9915	
AMG386 vs. AMG386 + Zaltrap	0,6402	
Zaltrap vs. AMG386 + Zaltrap	0,8217	
**Figure 4 H (CD3)**		
one-way ANOVA (Tukey's post test)		

control vs. AMG386	0,1972	
control vs. Zaltrap	< 0,0001	
control vs. AMG386 + Zaltrap	0,3494	
AMG386 vs. Zaltrap	0,065	
AMG386 vs. AMG386 + Zaltrap	0,9995	
Zaltrap vs. AMG386 + Zaltrap	0,213	
**Figure 4 H (Ly6G)**		
one-way ANOVA (Tukey's post test)		
control vs. AMG386	0,6334	
control vs. Vasculotide + Zaltrap	0,916	
control vs. Zaltrap	0,1256	
AMG386 vs. Vasculotide + Zaltrap	0,3997	
AMG386 vs. Zaltrap	0,0257	
Vasculotide + Zaltrap vs. Zaltrap	0,5399	
**Figure 4 I**		
	log-rank	wilcoxon
control vs AMG386	< 0,0001	< 0.0001
control vs aflibercept	< 0,0001	< 0,0001
control vs AMG386+aflibercept	< 0,0001	< 0,0001
aflibercept vs AMG386 + aflibercept	< 0,0001	< 0,0001
**Figure 4 K (CD206)**		
one-way ANOVA (Tukey's post test)		
Control vs. AMG386	0,0424	
Control vs. Aflibercept	0,4676	
Control vs. Aflibercept + AMG386	0,0751	
AMG386 vs. Aflibercept	0,8121	
AMG386 vs. Aflibercept + AMG386	0,9013	
Aflibercept vs. Aflibercept + AMG386	0,572	
**Figure 4 K (ratio)**		
one-way ANOVA (Tukey's post test)		

Control vs. AMG386	0,0528	
Control vs. Aflibercept	< 0,0001	
Control vs. Aflibercept + AMG386	< 0,0001	
AMG386 vs. Aflibercept	0,1254	
AMG386 vs. Aflibercept + AMG386	0,0011	
Aflibercept vs. Aflibercept + AMG386	0,1251	
**Figure 4 L**		
one-way ANOVA (Tukey's post test)		
Control vs. AMG386	0,0146	
Control vs. Aflibercept	0,1278	
Control vs. Aflibercept + AMG386	0,1872	
AMG386 vs. Aflibercept	0,5303	
AMG386 vs. Aflibercept + AMG386	0,4028	
Aflibercept vs. Aflibercept + AMG386	0,9945	
**Figure 5 D**		
Kruskal-Wallis (Dunn's post test)		
treatment-naive GBM vs. S / RTx / CTx	> 0,9999	
treatment-naive GBM vs. S / RTx / CTx / Bev	0,0296	
S / RTx / CTx vs. S / RTx / CTx / Bev	0,2799	
**Figure 5 G**		
Kruskal-Wallis (Dunn's post test)		
treatment-naive GBM vs. S / RTx / CTx	> 0,9999	
treatment-naive GBM vs. S / RTx / CTx / Bev	0,0268	
S / RTx / CTx vs. S / RTx / CTx / Bev	0,6731	
**Figure 5 H**		
Kruskal-Wallis (Dunn's post test)		
treatment-naive GBM vs. S / RTx / CTx	0,0352	
treatment-naive GBM vs. S / RTx / CTx / Bev	< 0,0001	
S / RTx / CTx vs. S / RTx / CTx / Bev	> 0,9999	

**Figure 6 C**		
Kruskal-Wallis (Dunn's post test)		
treatment-naive GBM vs. S / RTx / CTx	0,033	
treatment-naive GBM vs. S / RTx / CTx / Bev	< 0,0001	
S / RTx / CTx vs. S / RTx / CTx / Bev	> 0,9999	
**Figure 6 E**		
Kruskal-Wallis (Dunn's post test)		
treatment-naive GBM vs. S / RTx / CTx	> 0,9999	
treatment-naive GBM vs. S / RTx / CTx / Bev	0,0044	
S / RTx / CTx vs. S / RTx / CTx / Bev	0,3274	
**Figure 6 H**		
Kruskal-Wallis (Dunn's post test)		
treatment-naive GBM vs. S / RTx / CTx	> 0,9999	
treatment-naive GBM vs. S / RTx / CTx / Bev	< 0,0001	
S / RTx / CTx vs. S / RTx / CTx / Bev	0,1153	
**Expanded View Figure 1 C**		
Kruskal-Wallis (Dunn's post test)		
Low grade glioma vs. Anaplastic astrocytoma	> 0,9999	
Low grade glioma vs. Glioblastoma	0,0001	
Anaplastic astrocytoma vs. Glioblastoma	< 0,0001	
**Expanded View Figure 1 D**		
Kruskal-Wallis (Dunn's post test)		
NAGM vs. NAWM	0,2833	
NAGM vs. Infiltration zone	< 0,0001	
NAGM vs. Tumor center	< 0,0001	
NAWM vs. Infiltration zone	0,8571	
NAWM vs. Tumor center	< 0,0001	
Infiltration zone vs. Tumor center	0,0004	

**Expanded View Figure 3 B**		
2-way ANOVA		
day 5		
Control vs. Zaltrap	> 0,9999	
Control vs. AMG386	> 0,9999	
Control vs. AMG386+Zaltrap	> 0,9999	
Zaltrap vs. AMG386	> 0,9999	
Zaltrap vs. AMG386+Zaltrap	> 0,9999	
AMG386 vs. AMG386+Zaltrap	> 0,9999	
day 12		
Control vs. Zaltrap	0,9366	
Control vs. AMG386	0,984	
Control vs. AMG386+Zaltrap	0,9286	
Zaltrap vs. AMG386	0,9978	
Zaltrap vs. AMG386+Zaltrap	> 0.9999	
AMG386 vs. AMG386+Zaltrap	0,9969	
day 19		
Control vs. Zaltrap	< 0,0001	
Control vs. AMG386	0,003	
Control vs. AMG386+Zaltrap	< 0,0001	
Zaltrap vs. AMG386	0,6217	
Zaltrap vs. AMG386+Zaltrap	0,9974	
AMG386 vs. AMG386+Zaltrap	0,7294	
**Expanded View Figure 3 C**		
one-way ANOVA		
Control vs. AMG386	0,244	
Control vs. Aflibercept	0,5311	
Control vs. Afibercept+ AMG386	0,0039	
AMG386 vs. Aflibercept	0,9378	
AMG386 vs. Afibercept+ AMG386	0,2402	
Aflibercept vs. Afibercept+ AMG386	0,0787	
