# Supplementary material for: Endothelial cell‐derived angiopoietin‐2 is a therapeutic target in treatment‐naive and bevacizumab‐resistant glioblastoma
Source: EMBO Mol Med. 2015 Dec 14;8(1):39–57. doi: 10.15252/emmm.201505505 (PMC4718155; doi:10.15252/emmm.201505505)
Supplement: Supplementary file 2 — Expanded View Figures PDF [file EMMM-8-39-s002.pdf]

Expanded View Figures

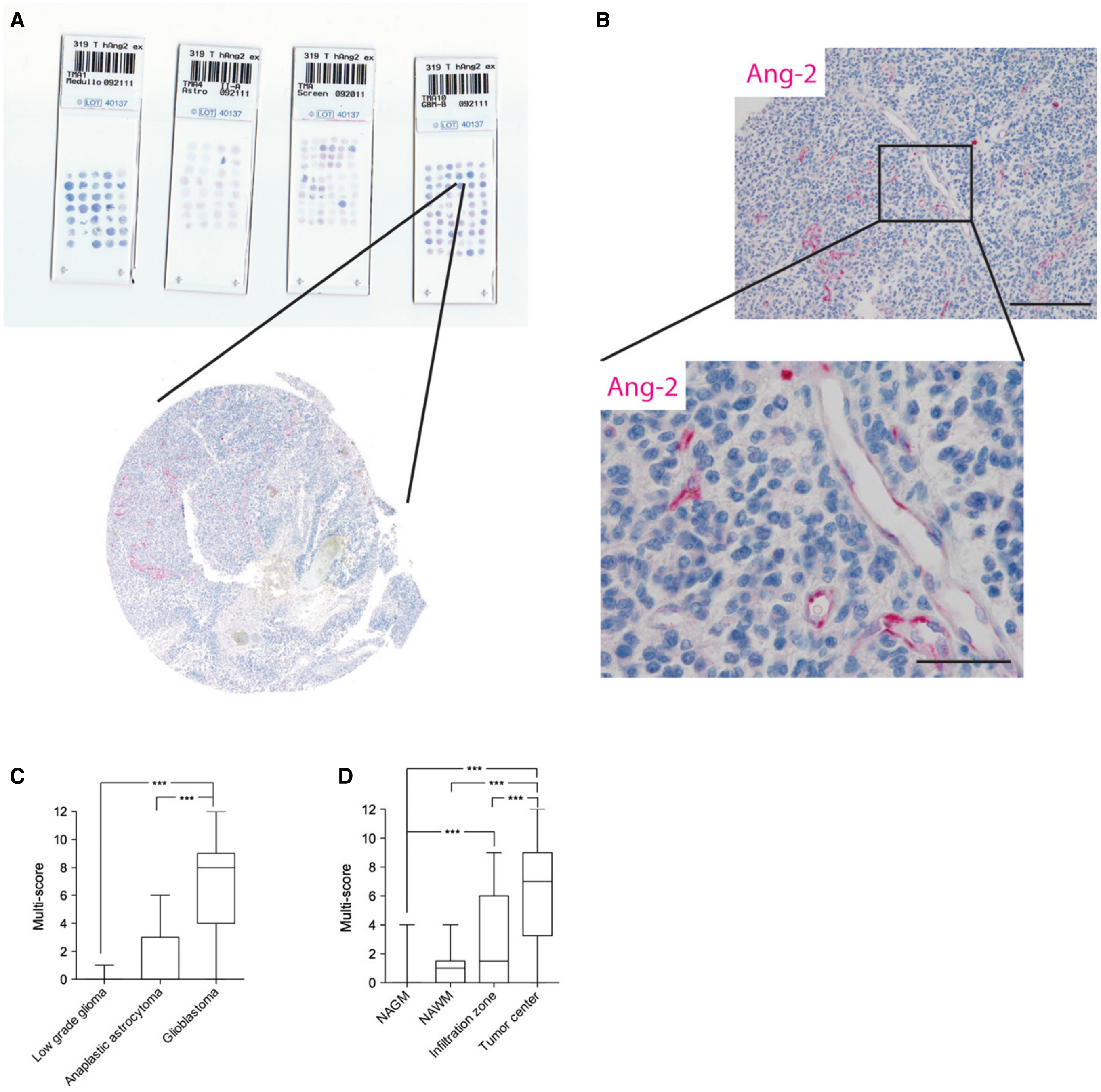

Figure EV1. Application of tissue microarrays (TMAs) for the analysis of human glioma samples.

A, B Biopsies of 303 human gliomas (WHO grade II, n = 16, WHO grade III, n = 35, and WHO grade IV, n = 252) were spotted on microscope slides (A) and processed for automated anti-Ang-2 immunohistochemistry (Ventana Benchmark platform) (B). Scale bar (B): 200 μm, inset: 50 μm.

C Ang-2 expression was assessed by applying a semiquantitative scoring system (Harter *et al*, 2010).

D Spatial expression of Ang-2 in brain specimens from glioblastoma patients was scored in normal-appearing gray matter (NAGM) (n = 48), normal-appearing white matter (NAWM) (n = 18), infiltration zone (n = 39), and tumor center (n = 62).

Data information: In (C, D), for statistical analysis, Kruskal–Wallis test (followed by Dunn's post-test) was applied. ***$P < 0.005$. Whisker Box plots displaying median, 25–75th percentile, upper and lower quartile.

© 2015 The Authors

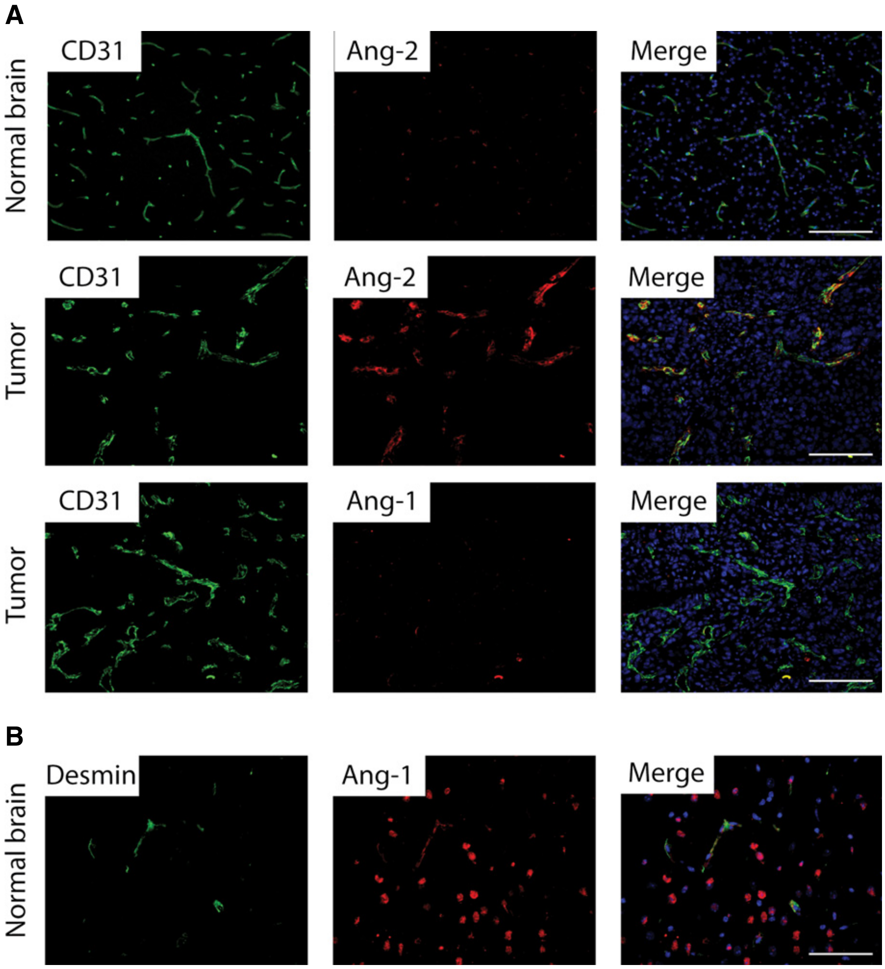

Figure EV2. Angiopoietin expression in mouse brain and GL261 tumors.

A, B Ang-2 and Ang-1 (red) immunofluorescence co-staining with anti-CD31 (green) was assessed in GL261 brain tumors (A). Ang-2 is not detectable in normal brain (A). Ang-1 (red) and desmin (green) double-immunofluorescence staining in normal mouse brain (B). Scale bars: 100 μm.

© 2015 The Authors

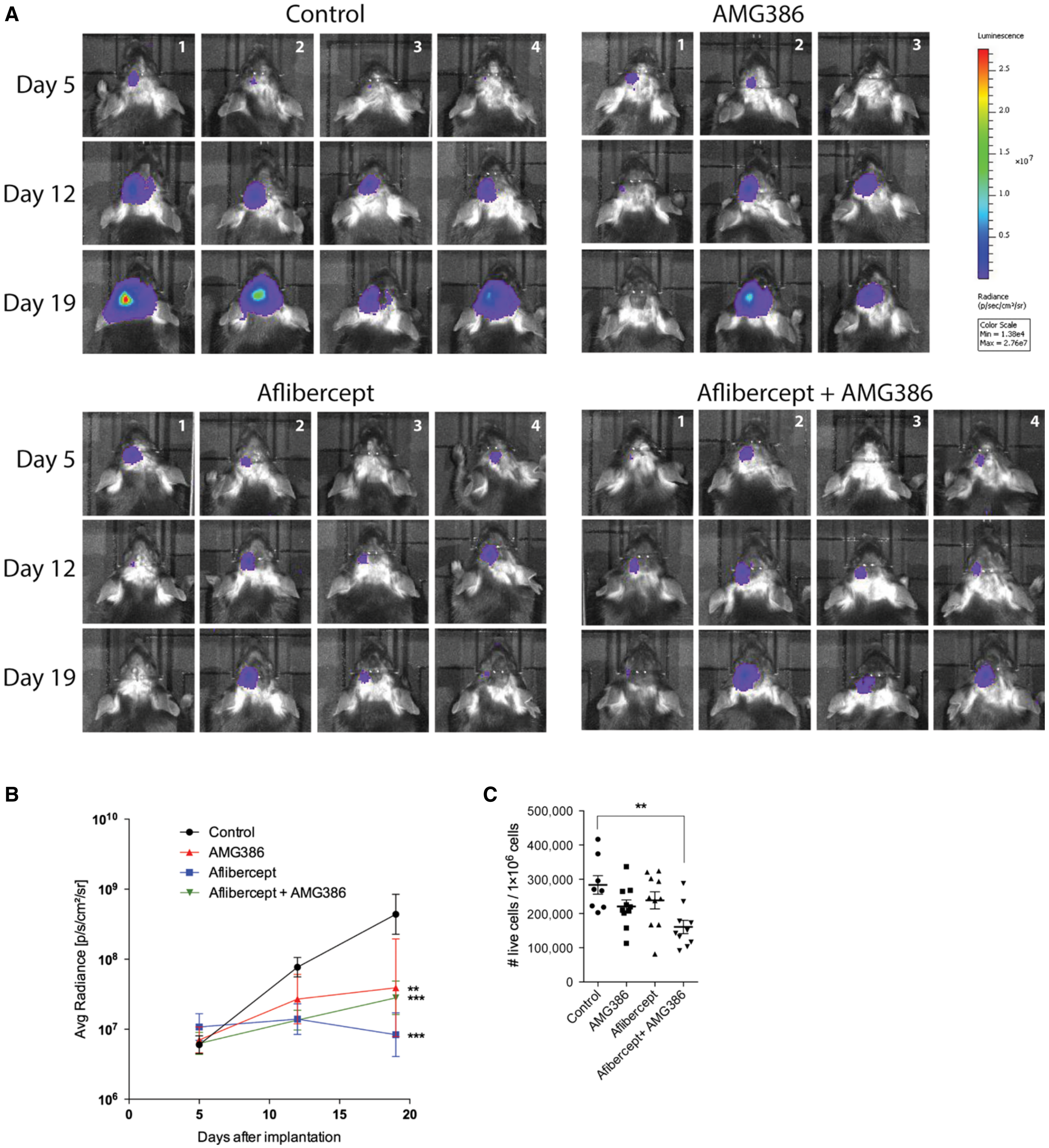

Figure EV3. Noninvasive imaging of GL261 tumor growth following anti-angiogenic therapy.

A, B Bioluminescence imaging of GL261-luc glioma cells on day 5, 12, and 19 after surgical implantation following AMG386, aflibercept, and combination treatment (A), and corresponding quantitative analysis (B). (Control *n* = 4; AMG386 *n* = 4; aflibercept *n* = 3; AMG386 + aflibercept *n* = 4.)

C Flow cytometric analysis of DAPI-negative live cells in mouse Gl261 brain tumors following anti-angiogenic therapy (control *n* = 4; AMG386 *n* = 4; aflibercept *n* = 5; AMG386 + aflibercept *n* = 5).

Data information: One-way (C) and two-way (B) ANOVA followed by Tukey post-test were performed for statistical analysis, $^{**}P < 0.01$, $^{***}P < 0.005$. Data are mean ± SEM (B), mean ± SD (C).

 © 2015 The Authors

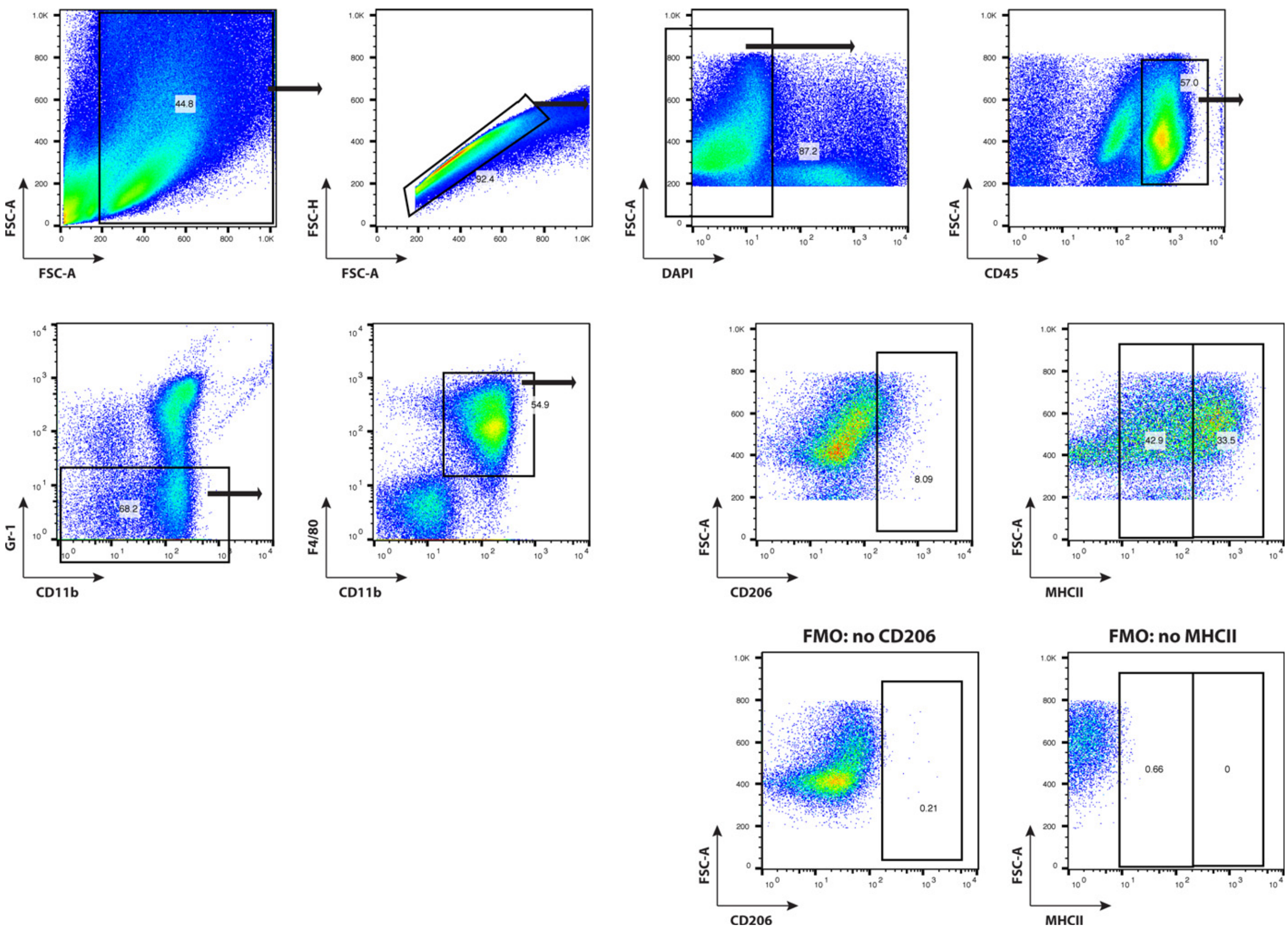

Figure EV4. Flow cytometry of brain tumor-infiltrating macrophages.
Gating strategy to identify brain tumor-infiltrating macrophages is displayed. M2-polarized, pro-angiogenic macrophages are identified as $CD45^{Hi}CD11b^{+}Gr\text{-}1^{-}F4/80^{+}CD206^{+}$. M1-polarized macrophages are identified by co-expression of MHC class II ($MHCII^{Hi}$) on $CD45^{Hi}CD11b^{+}Gr\text{-}1^{-}F4/80^{+}$ TAMs.

© 2015 The Authors
