# Supplementary material for: Endothelial cell‐derived angiopoietin‐2 is a therapeutic target in treatment‐naive and bevacizumab‐resistant glioblastoma
Source: EMBO Mol Med. 2015 Dec 14;8(1):39–57. doi: 10.15252/emmm.201505505 (PMC4718155; doi:10.15252/emmm.201505505)
Supplement: Supplementary file 3 — Review Process File [file EMMM-8-39-s004.pdf]

EMBO Molecular Medicine

# Endothelial cell-derived Angiopoietin-2 is a therapeutic target in treatment-naive and Bevacizumab-resistant glioblastoma

Alexander Scholz, Patrick N. Harter, Sebastian Cremer, Burak H. Yalcin, Stefanie Gurnik, Maiko Yamaji, Mariangela Di Tacchio, Kathleen Sommer, Peter Baumgarten, Oliver Bähr, Joachim P. Steinbach, Jörg Trojan, Martin Glas, Ulrich Herrlinger, Dietmar Krex, Matthias Meinhardt, Astrid Weyerbrock, Marco Timmer, Roland Goldbrunner, Martina Deckert, Christian Braun, Jens Schittenhelm, Jochen T. Frueh, Evelyn Ullrich, Michel Mittelbronn, Karl H. Plate, and Yvonne Reiss

*Corresponding author: Yvonne Reiss, Goethe University Medical School*

**Review timeline:**

| | |
|---|---|
| Submission date: | 05 June 2015 |
| Accepted: | 03 October 2015 |

*Editor: Céline Carret*

**Transaction Report:**

No Review Process File is available with this article, as the authors have chosen not to make the review process public in this case
